# Methoxyfuranocoumarins of Natural Origin–Updating Biological Activity Research and Searching for New Directions—A Review

**DOI:** 10.3390/cimb46010055

**Published:** 2024-01-19

**Authors:** Magdalena Bartnik

**Affiliations:** Department of Pharmacognosy with Medicinal Plants Garden, Medical University of Lublin, Chodźki 1 Street, 20-093 Lublin, Poland; magdalenabartnik@umlub.pl

**Keywords:** methoxyfuranocoumarins (MFCs), biological activity, secondary metabolites, plant drugs, isolated compounds activity, molecular mechanism of action, non-UV-activated molecules, bioavailability of MFCs

## Abstract

Plant secondary metabolites, including furanocoumarins, have attracted attention for decades as active molecules with therapeutic potential, especially those occurring in a limited number of species as evolutionarily specific and chemotaxonomically important. The most famous methoxyfuranocoumarins (MFCs), bergapten, xanthotoxin, isopimpinellin, phellopterin, byakangelicol, byakangelicin, isobergapten, pimpinellin, sphondin, as well as rare ones such as peucedanin and 8-methoxypeucedanin, apaensin, cnidilin, moellendorffiline and dahuribiethrins, have recently been investigated for their various biological activities. The α-glucosidase inhibitory activity and antioxidant potential of moellendorffiline, the antiproliferative and proapoptotic properties of non-UV-activated bergapten and xanthotoxin, the effect of MFC on the activity of tyrosinase, acetyl- and butylcholinesterase, and the role of these compounds as adjuvants in anticancer and antibacterial tests have been confirmed. The anticonvulsant effects of halfordin, the antidepressant effects of xanthotoxin, and the antiadipogenic, neuroprotective, anti-amyloid-β, and anti-inflammatory (via increasing SIRT 1 protein expression) properties of phellopterin, as well as the activity of sphondin against hepatitis B virus, have also attracted interest. It is worth paying attention to the agonistic effect of xanthotoxin on bitter taste receptors (TAS2Rs) on cardiomyocytes, which may be important in the future treatment of tachycardia, as well as the significant anti-inflammatory activity of dahuribiethrins. It should be emphasized that MFCs, although in many cases isolated for the first time many years ago, are still of great interest as bioactive molecules. The aim of this review is to highlight key recent developments in the study of the diverse biological activities of MFCs and attempt to highlight promising directions for their further research. Where possible, descriptions of the mechanisms of action of MFC are provided, which is related to the constantly discovered therapeutic potential of these molecules. The review covers the results of experiments from the last ten years (2014–2023) conducted on isolated natural cMFCs and includes the activity of molecules that have not been activated by UV rays.

## 1. Introduction

**Figure 1:**
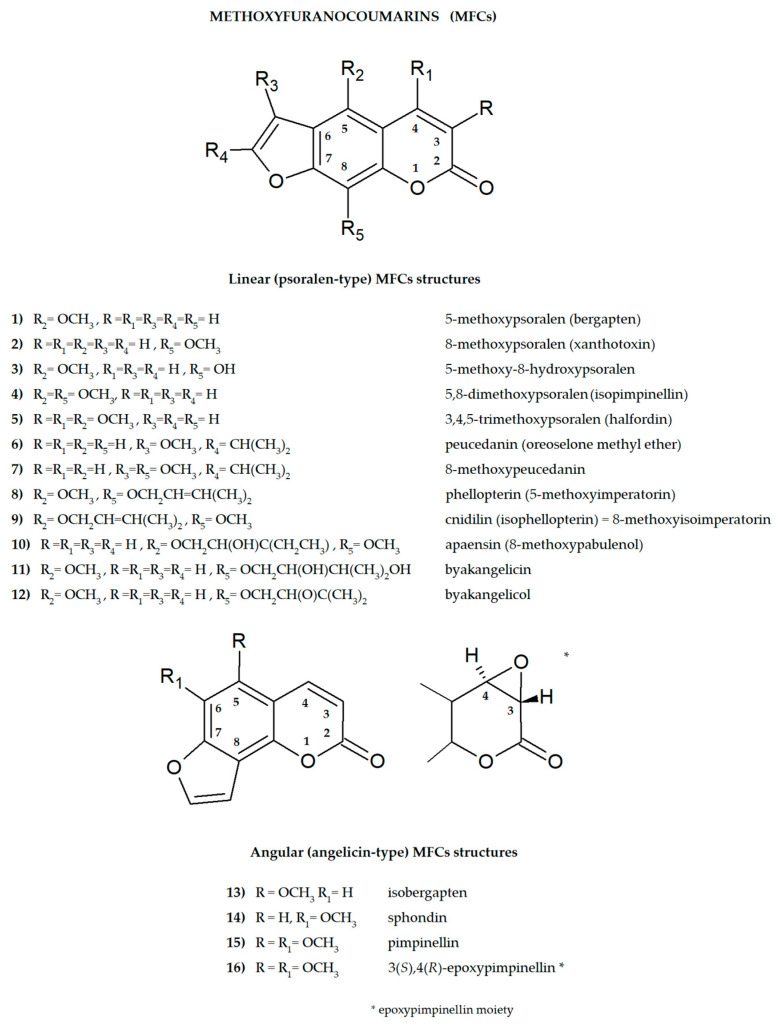
The main structures of linear (psoralen-type) and angular (angelicin-type) methoxyfuranocoumarins (MFCs), the subject of this review (ACD/ChemSketch–Freewere).

Furanocoumarins are secondary plant metabolites known to be elements of the plant defense system involved in the response to environmental factors: stressors, e.g., predators or parasites and various conditions such as lack of water, soil composition, salinity, and many others [1,2]. As active ingredients, they affect animals and humans, demonstrating various biological properties. For this reason, plants containing these molecules have been used as natural remedies in traditional medicine for centuries and are now the subject of modern drug development strategies. All of this was summarized recently in many important review articles [3,4,5,6,7,8]. The basic coumarin molecule has two six-membered rings fused together, of which one is a benzene ring and the other contains an alkene and ester functional group. Methoxyfuranocoumarins (MFCs), which are the subject of this review article, are a specific group of plant secondary metabolites, most often found and isolated from such botanical families as Apiaceae, Rutaceae, Moraceae and Fabaceae [9]. These specialized natural compounds have a benzo-α-pyrone system conjugated to the furan ring at the C-6/C-7 or C-7/C-8 position, forming linear (psoralen type) or angular (angelicin type) furanocoumarin structures, respectively (Figure 1) [3].

The furanocoumarin biosynthetic pathway has been the subject of research for decades. The first experiments with radioactively labeled precursors led to the identification of subsequent intermediate metabolites, indicating a very similar biosynthetic pathway in the species studied [10,11]. Briefly, the prenylation of 7-hydroxycoumarin, the so-called umbelliferone [CAS 93-35-6] (produced by the action of dioxygenase from p-coumaroyl-CoA), leads to linear and angular MFCs. It should be emphasized that this is the first stage of the biosynthesis of a large number of compounds performing defense functions in plants [10,11]. Prenylation of umbelliferone (due to the action of the prenyltransferase enzyme) to demethylsuberosin [CAS 21422-04-8] (alkylation at the C-6 position), which is oxidatively attacked at the double bond of the prenyl substituent by a P450-type enzyme, is producing (−) marmesin [CAS 495-32-9] as the product of this step in biosynthesis. The second P450 enzyme, psoralen synthase, stereospecifically affects the C-3′ hydrogen atom of the furan moiety, yielding a benzyl radical which undergoes β-cleavage, producing acetone, water and psoralen [CAS 66-97-7] as final products [12]. The sequence of oxidation and alkylation of psoralen results in the formation first of xanthotoxol and bergaptol and then of methoxylated furanocoumarins (*O*-methyltransferase enzyme) such as bergapten, xanthotoxin and isopimpinellin.

**Figure 2:**
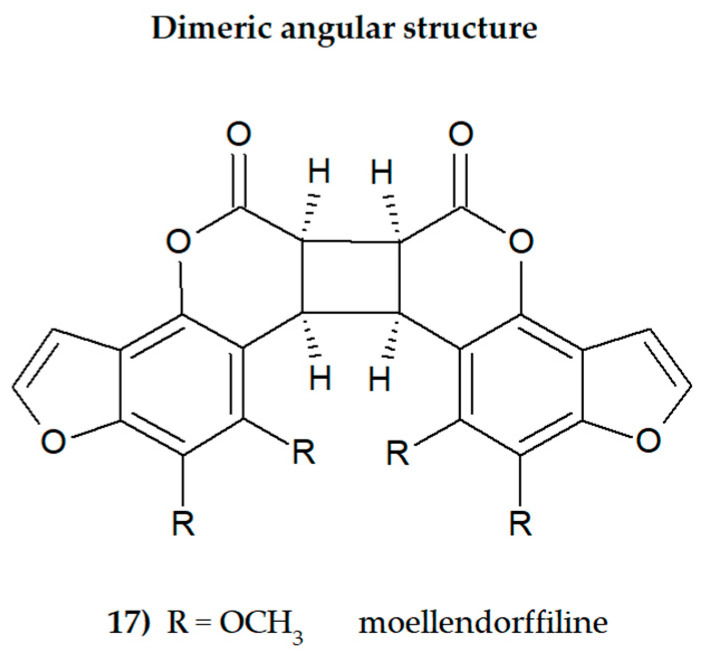
The structure of dimeric MFC, moellendorffiline (ACD/ChemSketch–Freewere.

**Figure 3:**
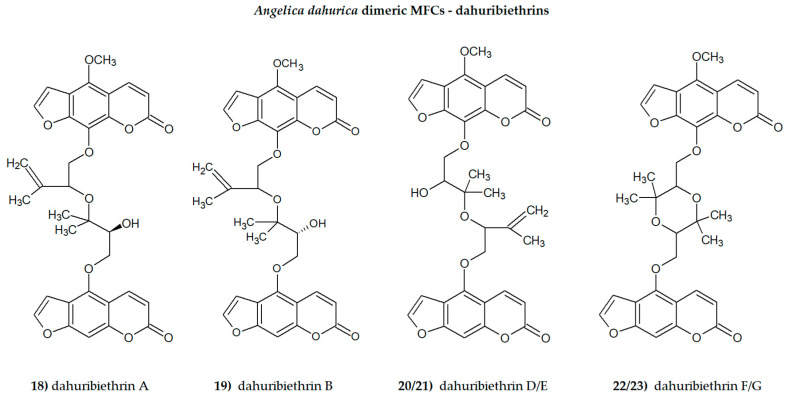
The dimeric *Angelica dahurica* MFCs, dahuribiethrins (ACD/ChemSketch–Freewere).

The presence of one or more methoxyl (-OCH_3_) groups (at C-3, -4, -5, -6, -7, or -8 positions) is important for their biological activity. MFCs may also possess hydroxyl, alkyl (methyl, propyl, isopropyl, butyl), or prenyl (3-methylbut-2-en-1-yl) moiety, and in some cases can be epoxidized. The structures of some MFCs, such as molendorffiline (a rare furanocoumarin formed by dimerization of pimpinellin) (Figure 2) and dahuribiethrins (A, B, D–F) (Figure 3), are dimers of angular and linear MFCs, respectively [13,14].

It was found that all these compounds are characterized by specific biological activity.

New methodologies for combining in silico molecular docking studies and then verifying the results in in vitro or in vivo studies constitute an interesting strategy for assessing the biological activity of MFCs [15]. Since psoralen derivatives have often been used after UV-A activation, which resulted in an increase in their phototoxicity, testing the biological activity of molecules not exposed to UV rays is an interesting approach in assessing the antiproliferative and antimigratory effects of these compounds, especially xanthotoxin or bergapten [16,17], against selected tumour cell lines. The synergistic effect of MFCs as adjuvants used with conventional drugs in anticancer treatment or as compounds that restore the sensitivity of resistant bacterial strains to antibiotics is also an important aspect of the potential use of these molecules in medicine [18,19]. The central nervous system is an important target of MFCs, and their antidepressant, anticonvulsant, and acetylcholinesterase inhibitory effects have recently been the subject of intense research [20,21].

The aim of this review is to present key findings from recent studies on the biological activity of MFCs and, where possible, to identify their mechanisms of action and provide insight into the relationship between their structure and biological activity. The goal of the article is also to draw attention to new directions that have appeared in MFC research over the last decade, as well as to show new perspectives for future challenges.

## 2. Materials and Methods

The articles used to prepare this review manuscript were collected from scientific databases such as SciFinder CAS, Scopus, Web of Science Clarivate, ScienceDirect, Medline, and PubMed. The collected data represent the results of research in this area in the recent ten years (2014–2023). The following keywords (and their combinations) were used to collect data: “methoxyfuranocoumarin/-s”; “bergapten”; “isobergapten”; “xanthotoxin”; “isopimpinellin” “pimpinellin”; “sphondin” “peucedanin”; “8-methoxypeucedanin”; “phellopterin”, “moellendorffiline”; “cnidilin” “byakangelicin”, “byakangelicol”, “halfordin” and “biological activity”, “mechanism of action”, “in vitro”; “in vivo”; “biological research”; “molecular mechanism”, “therapeutic use”.

The collected literature was organized in a way as to present new research aspects of the biological activity of the studied molecules, with particular emphasis on the molecular mechanisms of their action. The topic of the review article was to present research on the bioactivity of molecules not activated by UV rays.

## 3. General Aspects of MFC Studies

### 3.1. Studies of the Active Substituents in the MFC Structure

When attached to the benzene ring at the *para* position, a methoxyl group is classified, according to Hammett’s equation (developed in 1935), as electron-donating, but at the *meta* position it is classified as an electron-withdrawing group (in the *ortho* position, steric effects may cause the same trend as that of the *para* position) [19]. Hammett’s equation covers chemical reactivity, spectroscopy, and other physical properties, and even the biological activity of the drugs; however, it has some limitations.

As an example, for *meta*- and *para*-substituted aromatic compounds, inconsistency may be expected whenever the opportunity arises for strong electron delocalization between the substituent and the reaction sites [22].

In their recent work, Türker [23] discussed the MFC isomers with a single methoxy substituent attached to the psoralen backbone in various positions. This work, based on the density functional treatment (DFT), brings an interesting insight into the interactions of the methoxy groups (substituted at C-3, -4, -5, -6, -7, or -8 position) with heterocyclic oxygen, and with the carbonyl group in the tested molecules. The positional variations of the methoxy substituent in psoralen backbone result in electronically stable as well as thermally favourable isomers in all the studied cases. They all have exothermic heat of formation values, and the rings are found highly aromatic (especially isomers substituted at C-8 and C-5 positions on the benzoic ring) [23]. It is found that the heteroatoms in the rings have different extents of opposing and assisting effects on the net electron flow effect from the methoxy group. In the considered structures, lactone moiety acts as an electron-attracting, whereas the methoxy group acts as the electron-donating one, and the lactone carbonyl moiety embedded in the psoralen backbone attracts electrons mesomerically [23].

Hydroxylation of coumarin (attaching the -OH group to the carbon atom of the coumarin molecule) increases its solubility in water. In the human body, this mechanism facilitates the conjugation of the resulting hydroxyl derivative with glucuronide or sulfate metabolites and then its excretion from the body [24].

The double bond between C-3 and C-4 of the furanocoumarin molecule (as in the case of 3(*S*),4(*R*)-epoxypimpinelin—Figure 1) can be epoxidized. The epoxidized form of coumarin is unstable in the cellular environment and is therefore usually quickly eliminated/detoxified. It is also suspected that it may cause hepatotoxicity by itself or through its subsequent metabolites [25].

Prenylation is the conjugation reaction of the isoprenoid chain with various molecules, especially proteins. Prenyl groups have been shown to be important for protein–protein binding through a specialized prenyl-binding domain that may be important for enzymatic activity. There are also several groups of prenylated polyphenolic compounds in plants, including flavonoids, phloroglucinols, xanthones and coumarins [26]. Prenyl (3-methylbut-2-en-1-yl) moiety occurs in the structures of many MFCs and can be found as the simple 3-methylbut-2-en-1-yl unit, but is often found in the form of the corresponding epoxide or in an oxidized form. The prenyl group brings to the MFC structure the ability to interact with various molecules, especially with the active sites of enzymes, and offers these compounds new bioactive potential.

The previously discussed effects influence the biological activity of MFCs at a molecular level. It would be interesting to investigate the relationship between different MFC substituents and interactions within molecules containing methoxy (more than one) and prenyl functional groups, as well as both groups simultaneously. Such studies aimed at determining interactions within more complex MFC molecules could provide additional information useful for assessing their biological potential.

### 3.2. Bioavaiability Studies of MFCs

Bioavailability is related to the speed and the quantity of a drug appearing in the blood after a given dose, and many bioavailability studies involve determining the concentration of the ingested/injected active substance in blood or urine [5]. An important factor influencing the bioavailability and distribution of MFCs in the body is hydrophobicity, expressed in logP values (octanol/water partition coefficient). MFCs are mostly lipophilic and therefore have limited water solubility, which reduces their biological potential. On the other hand, as lipophilic substances, MFC diffuse well into the cell [27]. There are few studies concerning bioavailability of MFCs available, and there is still need to profoundly study the behavior of these molecules in the biological fluids of the body and membrane permeability investigations. The results of experiments exploring Parallel Artificial Membrane Permeability (PAMPA) models to predict transcellular passive absorption of MFC (bergapten and xanthotoxin) were recently published [28,29]. Using a hexadecane membrane, Petit et al. [28] studied the passive intestinal absorption of crude plant extracts of various compositions, including the extract of *Angelica archangelica* containing furanocoumarins. *A. angelica* MFCs have a high potential to easily cross the gastrointestinal barrier via the transcellular route, and it was found that the presence of multicomponent mixtures does not affect the passive permeability of these compounds as single components [28]. In another study, Li and co-investigators [29] found that the absorption of monocomponents (bergapten and isopimpinellin were analyzed) is related to their physicochemical properties (e.g., logP values); however, the chemical compatibility of the mixture components may change the absorption of co-existing compounds. Also, in the case of other MFCs, the use of the PAMPA model may provide new information on the absorption of these compounds into the bloodstream.

### 3.3. Distribution and Metabolism of MFCs

Zhao and co-workers [30] studied the pharmacokinetics of coumarins (5-hydroxy-8-methoxypsoralen, bergapten, xanthotoxin, isopimpinellin, neobyakangelicol, byakangelicin and phellopterin) occurring in a lyophilized ethanol extract (70%) from Angelicae dahuricae radix (ADR; single dose 6.0 g/kg) in the plasma of male Sprague–Dawley (SD) rats after oral administration. It was found that the coexistence of ADR drug components can significantly alter the pharmacokinetic behavior of a single compound, improving its bioavailability and extending its time in systemic circulation. Due to the similar chemical composition and structure, when taking a mixture of compounds, a pharmacokinetic phenomenon was observed, leading to the creation of enterohepatic circulation in order to maintain the effective concentration of the substance in the plasma and leading to long-term pharmacological effectiveness [30].

The following pharmacokinetic parameters were calculated: T_max_—maximum concentration, T_1/2_—elimination half-lives, and AUC (Area Under the Curve), a crucial measure of the bioavailability of a drug after administration. After oral administration of ADR, all of the tested coumarins were absorbed from rat gastrointestinal tract and detected (LC–MS/MS) at 5 min in plasma. T_max_ values of phellopterin, xanthotoxin, byakangelicin, and bergapten, were 1.7, 2.0 h, 2.3 h, and 2.4 h, respectively. 5-Hydroxy-8-methoxypsoralen, byakangelicin, bergapten, and phellopterin showed steeper slopes at the last time points of the concentration-time curves, which may indicate non-linear pharmacokinetics of these compounds due to, among others, the saturation of components in the system [30].

Liao et al. [31] analyzed xanthotoxin, isopimpinellin, and bergapten (from *Cnidium monnieri* fruit extract) in male SD rat plasma. Additionally, 24 metabolites of bergapten (13 in vitro and 23 in vivo) were detected, including 15 phase I metabolites and 9 phase II metabolites. It was found that oxidation and glucuronide conjugation might be the main methabolic pathways of MFCs. However, monooxidation, dioxidation and oxirolysis were the major metabolic pathways of bergapten in rat liver microsomes. The main biotransformation pathways in vivo were hydrolysis, hydrogenation and glucuronide conjugation. The pharmacokinetics study of MFCs was profoundly investigated. All of the compounds were absorbed rapidly and had similar elimination rates. However, isopimpinellin, with C-5 and C-8 methoxy groups, was eliminated slower than bergapten (C-5) and xanthotoxin (C-8). T_1/2_ values for isopimpinellin, bergapten, and xanthotoxin were 5.69, 4.21, and 3.40 h, respectively, and T_max_ values for compounds were 3.0 h, 3.0 h, and 2.0 h, respectively. Analyzed MFCs were detected by the UHPLC-Q-TOF-MS method [31].

The distribution of MFCs (bergapten, xanthotoxin, isopimpinellin, byakangelicin, phellopterin, 2″*R*–neobyakangelicol, isobyakangelicol) in male SD rats after oral administration of ADR (75% ethanol) was studied by Zhang et al. [32]. The MFCs were distributed widely and rapidly, and they could be detected in all of the selected tissues. However, the concentrations of coumarins were obviously higher in kidney, liver, and stomach and lower in testis, brain, and muscle tissues. The stomach, liver, and kidney might be the main target organs of phellopterin and byakangelicin because in these organs the concentrations of these MFCs were obviously higher than in other tissues. The concentration of 2″*R*–neobyakangelicol was high in stomach and kidney, which suggested that these are the main target organs of this MFC. On the other hand, in other selected tissues, especially in testis tissue, distribution was relatively low. The concentrations of xanthotoxin in stomach and kidney were obviously higher than those in other tissues, and the next was heart, from which the elimination of xanthotoxin was slow. The high concentration of isopimpinellin and isobyakangelicol in stomach prompted the hypothesis that stomach might be the main target organ of the two compounds. It was concluded that the rapid distribution of byakangelicin compared to phellopterin could be associated with hydroxyl groups in the byakangelicin structure, and therefore higher water solubility, which increased in in vivo fluids [32]. Quantitative analysis of the compounds was performed using use ultra-performance liquid chromatographic–tandem mass spectrometry (UHPLC-MS/MS).

Qiu and co-investigators [33] analyzed (HPLC-MS/MS) the amount of MFCs (isopimpinellin, pimpinellin and bergapten) after oral administration of *Toddalia asiatica* L. (Rutaceae) root extract (5.2 mL/kg, which was equal to 10.73 mg/kg isopimpinellin; 39.50 mg/kg pimpinellin; 9.73 mg/kg bergapten;) in plasma, urine, and feces of male SD rats. All of these MFCs were rapidly absorbed from the gastrointestinal tract. It was found that the amounts of the pimpinellin, isopimpinellin and bergapten excreted from urine and feces were extraordinarily limited (<0.5%), indicating that the four analytes were principally excreted in the form of bile or as metabolites. T_max_ was 0.33 h for isopimpinellin and pimpinellin, and 0.50 h for bergapten; T_1/2_ were 1.43 h; 0.91 h and 0.77 h, respectively. The C_max_ ratios and doses of pimpinellin, isopimpinellin, and bergapten were 1004, 201.6, and 107.6 ng/L, respectively [33].

### 3.4. Gut Metabolism of MFCs by Human Microbiota

The human body is inhabited by a huge number of commensal microorganisms, which include bacteria, viruses, and fungi. The vast majority of them colonize the gastrointestinal tract (GIT) [34]. Intestinal microflora (the so-called microbiome) plays an important role in various mechanisms occurring in the human body, such as the maturation and development of the immune system, the central nervous system, the GIT system, and is also responsible for basic metabolic pathways. These microbiome/host interactions are an area of research that is constantly evolving and cannot be underestimated [35].

The study of biotransformation by human intestinal bacteria is essential to evaluate the effects of the bioactive compounds present in foods and in natural medicines, and it is of great interest due to the confirmed altered biological activity of metabolites [36]. In the recently conducted study, furanocoumarins (bergapten, xanthotoxin and byakangelicol, among others) isolated form *A. dahurica* roots were metabolized by a human fecal sample and each MFC was transformed by *Blautia* sp. (MRG-PMF1), bacterium responsible for intestinal *O*-demethylation (as confirmed previously in the study of gut metabolism of polymethoxyflavones) [37]. The gut microbial conversion of xanthotoxin and bergapten with MRG-PMF1 strain resulted in formation of xanthotoxol and bergaptol due to the methyl aryl ether cleavage by bacterial *O*-methyltransferase. As the result of the biotransformation of byakangelicol, which underwent *O*-demethylation and hydration, three metabolites were detected: new metabolite–desmethylbyakangelicol, byakangelicin and finally desmethylbyakangelicin (as confirmed by HPLC-DAD-MS).

As was found for the first time, prenylated furanocoumarins such as imperatorin and isoimperatorin were transformed by MRG-PMF1 to deprenylated compounds, finally producing xanthotoxol and bergaptol [36]. Previously, it was known that prenyl groups in plant-derived polyphenols can undergo only hepatic I phase metabolism (with Cyt P-450 enzymes). Xanthotoxol can be more readily removed from the body with urine excretion due to its increased solubility, which is considered as a detoxification mechanism. As was confirmed in the described study, the human intestinal bacterium *Blautia* sp. MRG-PMF1 can metabolize MFCs isolated from *A. dahurica* roots [36]. Biotransformation studies of other MFCs by human microbiome could be beneficial for better understanding the biotransformation pathways of these compounds in the human body, and it is an area of study which is worth to be explored extensively.

### 3.5. New Approaches for Evaluation of MFC Activity

To enhance bioavailability of MFCs, nanoformulations of coumarins were examined to explore the anticancer potential of these compounds [38]. It is known that the basis of nanoformulation studies is divided into two main categories: nanocarriers (nanoparticles) and guest molecules. These may have influence on the solubility and biocompatibility of guest molecules. Microemulsions (thermodynamically stable isotropic systems with small particles < 100 nm) of xanthotoxin and chitosan-derivative-coated xanthotoxin were tried and found to be an effective way for drug delivery into the skin in dermal carcinomas [39].

New suitable animal models, such as zebrafish (*Danio rerio*), are currently being investigated, especially to evaluate the activity of MFCs in central nervous system (CNS) disorders, focusing on anticonvulsant and antiseizure effects [21].

Unfortunately, three-dimensional (3D) cell models/organoids for assessing the bioactivity of MFCs are still not available, and it should be emphasized that this may be a new approach worth trying, as the test model seems to be similar to in vivo tests. Three-dimensional organoids and organs on a chip are rapidly evolving, and these techniques have been widely characterized [40,41]. For example, three-dimensional (3D) in vitro models of the human brain, such as organoids, bioprinted three-dimensional models of brain tissues or functionalized organoids, may be important in the study of both stages of development and pathological changes within this organ [42], taking into account specific aspects such as anatomy, interactions at the cellular level and gene expression. These new models may provide a useful tool for investigating human-specific phenomena that cannot be studied in animal models. This may be important when designing new drugs, including those of natural origin. Such 3D cellular models can be produced from tissues taken from the patient, which provides a personalized approach to explaining disease mechanisms and developing treatment methods [41].

An interesting aspect of the research is the assessment of the activity of MFCs as agonists of human taste receptors, especially the T2R family, which are found not only in the oral cavity but also throughout the body, as, for example, in the GIT, the respiratory tract, the immune system and in the central nervous system. It has been found that these receptors are multi-targeted at the molecular level and for various compounds interacting with them, which may be related to their promising therapeutic potential [43]. Some MFCs, especially xanthotoxin, have been found to be effective molecules binding to T2R and exerting biological activity [44]. This aspect of MFC activity deserves special consideration in planned future studies.

## 4. Biological Activity of the MFCs–Recent Directions in the Study

The biological activity of MFCs is discussed below, highlighting new research results regarding the effects of these molecules on microorganisms and animal and human tissues in in vitro and in vivo studies.

### 4.1. Antimicrobial Activity of MFCs

#### 4.1.1. Antiviral Activity

Infections caused by viruses are a real concern of healthcare worldwide. In their review article, Li et al. [45] summarized various coumarin compounds (including semi-synthetic derivatives) as antiviral agents. Phellopterin has been shown to display activity against herpes simplex virus-1 (HSV-1) when used alone and in a mixture with imperatorin, reducing HSV-1 replication by 3.01 log and 3.73 log, respectively. As found by Rajtar et al. [46], the presence of an isopentenyloxy moiety at the C-8 position significantly improves the activity of the tested coumarins.

In vitro studies examined the inhibitory effect of neuraminidase (an enzyme that is a glycoprotein on the surface of the influenza virus, closely related to its replication and transmission) of selected compounds contained in the *A. pubescens* root extract. Xanthotoxin and phellopterin (100 µg/mL) inhibited the influenza virus enzyme by 54.71% and 52.87%, respectively [47]. Affinity ultrafiltration technology combined with LC-MS/MS (AUFLC-MS) was used for screening of the neuraminidase inhibitors in *A. pubescens* extract.

The analysis of antiviral activity of sphondin (angular MFC) against hepatitis B virus (HBV) was performed by Ren et al. [48]. As was confirmed, sphondin preferentially bound to the HBx protein (HBV protein important to transcription, signal transduction, cell cycle progression and stability of the virus in the host) by residue Arg72, and it resulted in increased 26S proteasome-mediated degradation of this protein, and decreased recruitment of HBx to cccDNA (covalently closed circular DNA). Sphondin inhibited cccDNA transcription and expression of HBsAg (hepatitis B surface antigen). No toxicity of sphondin was found in primary human hepatocytes (PHHs) and in HepG2-NTCP cells (HepG2 cells infected with pCMV plasmid). The ELISA assay demonstrated that sphondin had no effect at albumin nor apolipoprotein secretion even at a concentration of 90 µM. The results were also confirmed in vivo using a mouse model of HBV infection [48].

#### 4.1.2. Antibacterial Activity

Xanthotoxin, bergapten, phellopterin and pimpinellin were isolated from *H. mantegazzianum* Sommier and Levier fruits and their antimicrobial activity was tested against bacteria and yeasts, determining MICs (minimum inhibitory concentrations) for each compound. Phellopterin was active against *Staphylococcus aureus* (MIC = 0.03 mg/mL) and *S. epidermis* and *Micrococcus luteus* (MIC = 0.25 mg/mL). In this study, the synergism of bergapten and angelicin was found. The mixture of these compounds shows strong activity against Gram-positive bacteria and fungi (*Bacillus cereus* MIC = 0.06 mg/mL; *S. aureus* and *Bacillus subtilis* MIC = 0.125 mg/mL) and yeasts *Candida parapsilosis* (MIC = 0.06 mg/mL) compared with bergapten alone. As was found, the presence of the isopenthenyl group in the furanocoumarin skeleton increases their lipophilicity and possibility to passage of the molecule through bacterial membrane to its target site. Phellopterin possesses this group at the C-5 position. Additionally, the C-8 position of methoxygroup in the phellopterin molecule may enhance its antibacterial activity [49].

In the study of Zuo et al. [50], it was suggested that double oxygenated substituents at C-5 and C-8 are necessary for antibacterial activity of linear furanocoumarins, such as in the case of isopimpinellin and phellopterin. The latter one has additionally a prenyl group, which increases lipophilicity, and therefore phellopterin was more active against bacterial strains than isopimpinellin.

Bergapten, xanthotoxin and isopimpinellin, from *Ferulago carduchorum* Boiss. and Hausskn. were isolated, and it was found that all of them have significant antimicrobial activity against *S. aureus* strains (MIC = 1.87; 3.75 and 3.75 mg/mL, respectively). Confirmed activity against Gram-positive bacteria was higher than those found against Gram-negative bacteria (e.g., *E. coli*) [51].

After using TLC-bioautography screening of twelve Iranian plants, antibacterial compounds were selected from *Heracleum persicum*. Isolated compounds (bergapten, xanthotoxin, isopimpinellin, isobegapten, sphondin, pimpinellin and moellendorffiline) were tested against *S. aureus* (ATCC 25923), *B. cereus* (PTCC), *Pseudomonas aeruginosa* (PTCC 1430) and a clinical isolate of *Salmonella enteritidis* using a broth microdilution method to determine MICs. Tested concentrations of all of the compounds were 1.0–0.006 mg/mL. Moellendorffiline was found to be the most potent antibacterial compound (MIC of 15.62; 31.25; 31.25 and 62.50 µg/mL against *S. enteritidis; S. aureus, B. cereus*, and *P. aeruginosa,* respectively). Pimpinellin exhibited significant activities against *S. enteritidis* (MIC = 31.25 µg/mL) and *S. aureus* (MIC = 62.50 µg/mL). Angular MFCs were found to be more potent than linear ones, and dimers were more active than monomers. Moellendorffiline (dimer of pimpinellin) shows highest antibacterial activity (first time reported in this study). When comparing isopimpinellin (linear) with pimpinellin, the angular isomer of MFC was four-fold stronger against *S. enteritidis* and two-fold stronger against *S. aureus* than linear isomer. However, the activity of both compounds against *B. cereus* and *P. aeruginosa* were similarly low, which suggests that antibacterial activity of MFCs is also species specific [14].

#### 4.1.3. Antifungal Activity

Song et al. [52] studied the antifungal properties and structure–activity relationships of isolated natural MFCs, xanthotoxin, byakangelicin, isopimpinellin, phellopterin, cnidilin and bergapten. In vitro antifungal activity was assessed in darkness to avoid potential phototoxic degradation of the tested compounds (the tested concentration was 30 µg/mL for each compound, and the inhibition rate was given in %). The tested fungal strains (*Sclerotinia sclerotiorum, Botrytis cinerea, Thanateforus cucumeris, Fusarium graminearum* and *Coletotrichum capsici*) are well-known plant pathogens; they were isolated from natural sources. It was found that xanthotoxin and isopimpinellin were active against *S. sclerotiorum* (78.8% and 76.8% inhibition, respectively, and EC50 = 14.6 µg/mL for xanthotoxin and 20.7 µg/mL for isopimpinellin). It was observed that the antifungal activity is influenced by the specific position of the substituents in the coumarin structure, especially the C-5 and C-8 substitution.

He et al. [53] investigated the mechanisms of antifungal activity of MFCs (bergapten, isopimpinellin, xanthotoxin, sphondin, pimpinellin and other compounds such as methoxy-substituted simple coumarins) isolated from *Cnidium monnieri* (L.) Cusson fruits in regard to five pathogenic fungi, *Magnaporthe oryzae*, *B. cinerea*, *F. graminearum*, *S. sclerotinum* and *Rhizoctonia solani.* Pimpinellin inhibited the above-mentioned fungi by 61%, 58%, 63%, 20%, and 67%. Xanthotoxin inhibited *R. solani* (88%), *F. graminearum* (63%), and *M. oryzae* (83%). Isopimpinellin inhibited *M. oryzae* by 64%, and bergapten was mainly active against *S. sclerotiorum* (34%). It was found that the presence of methoxy groups at C-8 and C-5 in the structure of furanocoumarin and the angular type of this structure are beneficial for the activity. The most active was, however, simple 4-methoxycoumarin (methoxy group at C-4), inhibiting the growth of *R. solani* by 100% and other species in the range of 45–81%. Based on gene expression studies, it was found that this compound affects the structure and function of peroxisomes and inhibits the β-oxidation of fatty acids, which finally causes cell death of the mycelium of *R. solani* [53].

#### 4.1.4. Nematicidal Activity

Pine wood nematode *Bursaphelenchus xylophilus* is the main pathogen of pine wilt disease (PWD), which destroys the population of these trees. MFCs (isopimpinellin, bergapten, and xanthotoxin) isolated from the roots of *C. monnieri* and *A. dahurica*, considered to be environmentally friendly plant-derived nematicides, were investigated by Feng et al. [54]. A strong nematicidal effect on *B. xylophilus* was found, affecting reproduction, egg hatching and feeding ability. Additionally, inhibition of acetylcholinesterase (AChE) and the Ca^2+^ ATPase of *B. xylophilus* was confirmed for all tested compounds. Inhibition of AChE activity results in ACh accumulation within synapses, excessive stimulation of cholinergic neurons, and finally increase in nematode mortality. ATPase, which is essential for Ca^2+^ homeostasis inside the cells, plays a key role in a wide spectrum of intracellular signaling processes. Both of these nematode enzymes were targets for MFCs acting as nematicide agents.

### 4.2. Anti-Inflammatory Activity of MFCs

Dimeric furanocoumarins dahuribiethrins A–G isolated from the roots of *A. dahurica* (Fish ex Hoffm.) Benth. Et Hook. were investigated as anti-inflammatory agents, and their inhibition of nitric oxide (NO) production in the LPS (lipopolysaccharide)-stimulated murine macrophage RAW264.7 cell line was evaluated. Dahuribiethrins B, D and E exhibited significant inhibition of NO (IC50 = 9.6; 8.8 and 9.2 µM, respectively) in the tested model compared with indomethacin (IC50 = 23.6 µM) [13].

Neutrophil-dominated inflammatory diseases and chronic inflammation are important health problems. The inflammation process recruits the neutrophils into the place of injury, and to avoid chronic inflammation these cells must be cleared by apoptosis or reverse migration. Investigation of isopimpinellin as an anti-inflammatory compound was conducted by Robertson et al. [55], confirming that isopimpinellin induces neutrophil apoptosis influencing caspase-3 activity in zebrafish in an in vivo model of inflammation. Neutrophil migration relies on the polarization of the cell, which is dependent on phosphatidylinositol 3-kinase (PI3K) signaling. It was found that isopimpinellin inhibited PI3K and reduced the migration of neutrophils towards the site of injury. The experiments suggest that MFC acts either directly on or upstream of PI3K, resulting in reduced phosphorylation of the pH domain of Akt to the plasma membrane and disruption of directional migration of neutrophils towards the wounds.

In the study of Li and Wu [56], coumarins isolated from the roots of *A. dahurica* were assessed on their anti-inflammatory potential in a histamine release test (as the allergic inflammation mediator which acts by binding into the H1 receptor) in basophilic leukaemia (RBL-2H3). Allergic inflammation was tested in vitro, after in silico modeling for searching of H1 receptor agonists. The measurement of levels of TNF-α, IL-1β and IL-4 inflammatory cytokines was conducted using ELISA. Also, the Luciferase reporter assay for NF-*ĸ*B (nuclear factor kappa-light-chain-enhancer of activated B cells) was measured. It was found that bergapten shows the greatest impact on the reduction of the histamin level in RBL-2H3 cells, followed by byakangelicin, isopimpinellin, byakangelicol and pimpinellin (the concentration of compounds was 20 µM). All of the tested compounds showed lowering activity levels of TNF-α, IL-1β and IL-4 and affecting the lowering Luciferase activity. As was concluded, the furan ring fusion at the C-6 and C-7 positions of the coumarin scaffold is necessary, the 5-methoxy group contributes greatly to reducing histamine release, and isopentane-derived groups in the molecule likely reduce histamine release as well. The aforementioned properties in the MFC structure are also beneficial to reduce TNF-α, IL-1β and IL-4 secretion. The tested MFCs inhibit the expression of inflammatory cytokine genes by the activation of NF-*ĸ*B. Additionally, the logP values of the tested compounds (1.71–4.04) confirm that these molecules can effectively permeate the cell membrane and be delivered to the binding sites.

It was confirmed that the interaction of epidermal keratinocytes with immune cells causes the initiation of atopic dermatitis (AD). Activation of STAT3 (a transcription factor involved in a broad spectrum of immune functions) by interleukin-4 (IL-4) causes inflammation and AD progression by promoting the transcription of TLSP (thymic stromal lymphopoietin involved in the inflammatory process) and IL-33 (affecting cytokine production) [57].

In the study of Chen and co-investigators [58], phellopterin was found to be active against two types of inflammation. This MFC reduces serum immunoglobulin E (IgE) levels and migration of eosinophils and mast cells in AD skin lesions. It was found that this in vitro effect is exerted by suppression of phosphorylation of the signal transducer and STAT3 at Tyr705, the expression of TSLP and IL-33 in in vitro studied human epidermal keratinocytes (HaCaT) and in vivo experiments in C57BL/6 male mice (threated with MC903 solution applied topically to the ear over 19 days to induce AD). The concentrations of phellopterin (0.5; 1.5 and 4.5 µg/mL) were mixed with 0.5% carbomer gel and applied topically (next 10 days). It was also confirmed that the application of phellopterin significantly reduced epidermis thickness and the number of eosinophils in the treated group.

In the human diabetic ulcer tissues, the low levels of SIRT1 (silence information regulator 1 protein) and high levels of IFN-γ (interferon γ) and ICAM-1 (intracellular cell adhesion molecule-1) were detected. In the study by Zhou et al. [59], it was found that phellopterin exerts an anti-inflammatory effect that accelerates the healing of diabetes-related wounds by upregulating SIRT1 and reducing ICAM-1 levels. Phellopterin promoted re-epithelization (keratinocyte proliferation) in vitro in an HaCaT cell IFN-γ-induced inflammatory model. These results were also confirmed in vivo in a streptozotocin-induced diabetic model in C57BL/6J mice, where phellopterin was used as an active molecule in a topically applied cream. Human diabetic ulcers were surgically introduced into the diabetic mice, and the expressions of IFN-γ, ICAM-1 and SIRT1 were studied before and after MFC treatment. Phellopterin application significantly shortened the wound healing time compared to the control group. However, when SIRT1 was knocked down, the effects of phellopterin were inhibited, confirming that the SIRT1 protein is an important target for this MFC [59].

### 4.3. Antioxidant Potential of MFCs

In MFC studies with *H. persicum*, moellendorffiline (dimeric MFC) showed significant antioxidant activity (IC50 = 0.2 µM) compared to butylated hydroxytoluene (IC50 = 0.1 µM) in the DPPH (2,2–diphenyl-1-picrylhydrazyl) radical scavenging test. The most effective antioxidants were found to be angular MFCs, especially with two methoxy groups at C-5 and C-6 positions simultaneously (pimpinellin; IC50 = 1.2 µM), and the most active of the MFCs was the mentioned dimeric compound. Linear MFCs were less active than angular ones. The location of the methoxy group at C-8 was important for activity in the DPPH assay, and xanthotoxin (methoxy at the C-8 position) was the most active of the linear MFCs (IC50 = 12.0 µM) [60].

DPPH radical scavenging activity of MFCs from *A. dahurica* roots was evaluated, and it was found that xanthotoxin (with the C-8 methoxyl group) inhibited the DPPH radical in 27%, and bergapten (C-5 methoxyl group) in only 11% (compared to 83% inhibition exerted by ascorbic acid as positive control). Interestingly, xanthotoxol (possessing the C-8 hydroxyl group) has inhibitory activity c.a. 66%, and a presence of the hydroxyl group was found to be important for this activity [61]. Isolated from *A. dahurica* roots, 5-methoxy-8-hydroxypsoralen exhibited moderate DPPH^•^ scavenging activity (EC50 = 45.24 µM) and strong ABTS^•+^ scavenging activity (EC50 = 17.86 µM) [62].

In another antioxidant assay it was confirmed that trimethoxyfuranocoumarin halfordin (methoxy groups at C-3, C-4, and C-5), isolated from *Melicope latifolia* (DC) T.G. Hartley (Rutaceae) bark, exerts high antioxidant activity in the β-carotene bleaching assay (88.48% of inhibition) [63].

### 4.4. MFCs as Therapy Adjuvants

Adjuvant is an ingredient in medicine that increases or modifies the activity of the other components/drugs. It has been found that furanocoumarins support the therapy of antibacterial and anticancer drugs, enabling the restoration of the sensitivity of bacterial strains to the drugs used, and are also useful in reducing the necessary dose and alleviating the side effects of drugs used in anticancer treatment. The mechanism of synergistic action of mixtures of compounds has not been clearly explained; therefore, in-depth research is necessary to effectively use this phenomenon in practice.

#### 4.4.1. Adjuvants in Antibacterial Treatment

Joshi et al. [64] reported that phellopterin two-fold potentiated the activities of ciprofloxacin (reduced the MIC of this drug from 8 to 4 µg/mL) against SA1199B (NorA which overexpress the *norA* gene that encodes the NorA efflux protein, responsible for the efflux of fluorquinolones) and erythromycin against SA-K2191 (MrsA, protein correlated to the efflux of macrolides), and this effect was correlated with P-glycoprotein inhibition by this MFC with IC_50_ of 32 µg/mL. The potentiating effect of MFCs on drugs against multidrug-resistant strains is an important topic, and it is worth to continue investigation to fight against clinical MRSA infections.

In the study by Zuo and co-workers [50], phellopterin and isopimpinellin were evaluated on their antimicrobial activity, focusing on the potential of these MFCs to restore the activity of conventional antimicrobials used against methicillin-resistant *Staphylococcus aureus* (MRSA) strains. To evaluate the activity of individual compounds isolated from the ether extract of the roots of the Chinese drug *Zanthoxylum nitidum* (Roxb.) D.C. (Rutaceae), the double broth microdilution method was used. The prenyloxy group of phellopterin was found to contribute more to anti-MRSA activity than the methoxy group of isopimpinellin (MIC90, µg/mL; 32 and 64, respectively). Additionally, compared to imperatorin and isoimperatorin (having only prenyl groups; MIC values > 1000 µg/mL), the methoxy group (oxidized substituents) at C-5 and C-8 was found to be essential for antibacterial activity. The prenyl group in phellopterin increases lipophilicity, thus facilitating passage through the microbial membrane, which was also previously found in another study [49]. The authors also assessed the synergy of the tested MFCs with various antibacterial drugs, such as gentamycin, chloramphenicol, fosfomycin, levofloxacin, minocycline, and vancomycin, and it was found that in the case of the combination of gentamycin/chloramphenicol/phellopterin, MIC was ≤8 ug/mL. Moreover, phellopterin and isopimpinellin restored the susceptibility of three of the four MRSA strains to levofloxacin and minocycline, respectively.

In the second study, from three MFCs, namely bergapten, xanthotoxin and isopimpinellin, isolated from the aerial parts of *Metrodorea mollis* and *Pilocarpus spicatus* (Rutaceae), only isopimpinellin with two methoxy groups (and which is therefore the most lipophilic) was active, four-fold reducing MIC for erythromycin, without affecting tetracycline and norfloxacin [19].

#### 4.4.2. Adjuvants in Anticancer Therapy

An important task in the drug discovery process is the attempt to use combined treatment of cancer with natural products and chemotherapy drugs. This is promising due to the synergistic effect, that occurs in some cases, which can lower drug doses and reduce the side effects of anticancer therapies, causing minimal toxicity to non-cancer host cells [18]. Recently, studies combining MFCs with anticancer drug 5-Florouracil (5FU) were conducted by Zhu et al. [65]. Bergapten, isolated from whole *Pleurospermum lindleyanum* plants, tested at concentrations of 100 µM and 200 µM, was confirmed to synergistically enhance the inhibitory effect of 5FU against human hepatocellular carcinoma cells (SMMC-7721) while showing no cytotoxicity against human embryonic kidney cell line (HEK293T).

#### 4.4.3. Adjuvants in Anticonvulsant Treatment

The anticonvulsant effect of isopimpinellin isolated from *P. sativa* was tested in the mouse model of maximal electroshock-induced (MES) tonic-clonic seizures by Łuszczki et al. [66]. Isopimpinellin was administrated in doses of 25 and 50 mg/kg; it was used alone or in compilations with borneol (monoterpenoid) and its influence on antiseizure potencies of different classes of antiseizure drugs (ASDs), such as carbamazepine (CBZ), phenytoin (PHT), phenobarbital (PB) and valproate (VPA), was tested. It was found that isopimpinellin administrated alone had no influence on the anticonvulsant potencies of ASDs. Moreover, isopimpinellin with VPA produced antagonistic interaction in the mouse model. However, when administrated with borneol and with PB or VPA, it produced additive interactions in the mouse model, and unfortunately it was also observed that borneol increased the total brain concentration of the tested ASDs.

#### 4.4.4. Modulation of Distribution and Bioactivity of Drugs in the Brain

Byakangelicin, one of the most active compounds of *A. gigas*, was studied in B57/BL6 mice (in an in vivo model of LPS-induced neuroinflammation and then subjected to ex vivo imaging) analyzing the effect of curcumin and byakangelicin/curcumin mixtures on inflammatory processes by measuring cytokine levels, tumor necrosis factor α, and interleukin-1β (TNF-α, IL-1β) in mouse brain homogenates and serum using ELISA [67]. Direct ex vivo fluorescence monitoring revealed that the levels of active drugs in the brain were effectively increased when co-administered with byakangelicin (umbeliferon 4.2-fold, curcumin 2.3-fold and doxorubicin 5.7-fold), without disturbing the blood–brain barrier. Additional accumulation of active substances reduced LPS-induced inflammation in the brain (there was a decrease in the level of pro-inflammatory cytokines; TNF-α and IL-1β), especially after injection of byakangelicin/curcumin. Byakangelicin was found to act as a modulator, enabling better accumulation of active compounds injected simultaneously and improving the therapeutic effect of the drugs.

### 4.5. Cytostatic Activity; Anti-Proliferative, Anti-Migrative and Pro-Apoptotic Activity of Non-UV-Activated MFCs

Apoptosis is controlled cell death, regulated by many factors and triggered by complex pathways, crucial for maintaining body homeostasis and essential for the anticancer effects of drugs without destroying normal cells. That is why so much effort is put into finding pro-apoptotic molecules of natural origin that effectively and precisely trigger apoptosis of cancer cells without causing necrotic effects.

Nur77 (also called NGFI-B or TR3), an orphan member of the nuclear receptor superfamily, plays a critical role in the regulation of the growth, survival, and apoptosis of cancer cells via the Nur77-Bcl-2- apoptotic pathway. In the study conducted by Zhou et al. [68], it was found that apaensin (isolated from *A. dahurica* roots) targets orphan nuclear receptor Nur77 through its unique activation of JNK and p38 MAPK and provides insight into the complex regulation of this pathway of apoptosis. It can effectively induce apoptosis in NIH-H460 lung cancer cells (known to be sensitive to the Nur77 dependent apoptotic effect). The number of apoptotic cells was 43.6% after a 6 h treatment of apaensin (10 μM); however, after only 3 h of treatment, significant effect was already observed, and apoptosis was induced by apaensin in a dose- and time-dependent manner. When cells were treated with apaensin, the distribution patterns of Nur77 (mainly nuclear in cells) and Bcl-2 (B-cell lymphoma 2 protein, resided predominantly in the cytoplasm) overlapped extensively in the cytoplasm. Nur77 might target mitochondria through its interaction with Bcl-2 (Nur77 converts Bcl-2 from an antiapoptotic to a proapoptotic molecule), which acts to activate Bax (bcl-like-protein 4; Bcl2-L-4). This activation was important for the apoptotic effect of apaensin [69].

The antiproliferative activity of MFCs (phelloterin, 5-methoxy-8-hydroxypsoralen, byakangelicol and byakangelicin; concentrations tested; 5–80 µM) from *A. dhurica* were evaluated in the MTT assay in HeLa (human cervical cancer), HepG2 (human hepatoblastoma) and MCF-7 (Human breast cancer) cell lines (5FU was used as the positive control) [62]. Phellopterin and 5-methoxy-8-hydroxypsoralen showed the highest inhibition on HepG2 cells (IC50 = 7.49 and 7.46 µM, respectively). The latter compound was also active against the HeLa cell line (IC50 = 13.48 µM). It was concluded that the prenyl or the prenyloxy groups at C-5 or C-8 and the hydroxy group at C-8 play an important role in the growth inhibition on these cancer cells.

The influence of bergapten, xanthotoxin, peucedanin and 8-methoxypeucedanin (concentrations: 1; 10, and 100 µM) isolated from *Peucedanum luxurians* Tamamsh. (combined LC/prepTLC) on apoptosis induction and immunomodulating activity in promyelocytic cells (HL60) and on peripheral blood lymphocytes (PBL) was recently studied [68]. Apoptosis and cell cycle distribution were evaluated using acridine orange assay and flow cytometry. Additionally, in TUNEL methodology, BrdUTP tests were used to confirm in situ labeling DNA presence of apoptotic cells. It was found that doses of 100 µM showed maximal effect after 48 h on the percentage cells in the G1 phase of the cell cycle (bergapten 47.3; xanthotoxin 44.2; peucedanin 58.6; 8-methoxypeucedanin 51.9%) compared to control (39.9%). Peucedanin and 8-methoxypeucedanin inhibited the cell cycle in the G1 phase after 24 h (69.1 and 66.1%, respectively, vs. control; 41.1%). The percentages of apoptotic HL60 cells were 11.5 and 14.9 (for bergapten and xanthotoxin, respectively) after 48 h (control 3.3%). To additionally examine these effects, the experiments on PHA (phytochemaglutynin)-stimulated PBL cells were treated with abovementioned compounds. The coumarins were added in the G1 cell cycle phase of these cells, and low or moderate apoptosis induction was observed, and no cell block was observed in the PBL cell cultures [68].

In other studies, investigations of non-UV-activated xanthotoxin and bergapten isolated by centrifugal partition chromatography from the *Ammi majus* L. fruit extract and the *Peucedanum tauricum* M. Bieb. fruit extract, respectively, were tested in in vitro studies on different human resistant cancer cell lines. As was found, these MFCs (6.25–100 µM) inhibited proliferation and migration of these cancer cells (tested in MTT and wound healing assays) in a dose-dependent manner. Independently, no toxicity was observed for these compounds in concentrations less than 100 µM (in normal human fibroblasts). MFCs exerted pro-apoptotic activity in tested cancer cell lines, and a detailed mechanism in most susceptible cell lines was elucidated [16,17].

Xanthotoxin inhibited the growth of several cancer cell lines, and the neuroblastoma (SK-N-AS) and metastatic colon cancer (SW620) cells were the most sensitive to this MFC (IC50 = 56.3 μM and 88.5 μM, respectively). Xanthotoxin reduced the phosphorylation of AKT^308^ (protein kinase B; phosphoroAkt-thr308). It was observed that non-UVA-activated xanthotoxin impairs the PI3K(phosphatidylinositol-3-kinase)/AKT signaling pathway and can inhibit the growth of neuroblastoma and colon cancer cells by induction of apoptosis via intrinsic and extrinsic pathways [16]. Activation of caspase-8, and caspases-9 and -3 was observed, as well as their influence on the expression of proteins Bcl-2 (decreased) and Bax (increased). In the case of bergapten, a similar mechanism was observed, and as for xanthotoxin, the effects were cell specific and dose dependent.

From the tested cell lines, osteosarcoma cell line Saos-2 was the most sensitive to bergapten (40.05 µM) compared with the six times less sensitive osteosarcoma HOS line. Moderate sensitivity of colon cancer cells (332 µM for HT-29 and 345.5 µM for SW620) was observed. The weakest effects in multiple myeloma cells RPMI (1272 µM) and U266 (1190 µM) were found. Bergapten was able to block the cell cycle in the G2 phase and trigger apoptosis, as was detected in flow cytometry. Caspase-8, -9, and -3 activation and alteration of Bcl2 and Bax proteins were observed as in case of xanthotoxin. The loss of mitochondrial membrane potential, decreased AKT phosphorylation, and impairment of the PI3KAKT signaling pathway were the mechanisms involved in the proapoptotic action of bergapten [17].

### 4.6. Cytochrome Inhibitory Activity and Related Drug and Xenobiotic Metabolism

#### 4.6.1. Inhibition of Melatonin Metabolism

Melatonin plays a considerable role in the regulation of circadian rhythms as an endogenous hormone, and nowadays it is also used as medicine. This compound is extensively metabolized and is prone to be affected by different extrinsic factors, among others also by simultaneous ingestion of furanocoumarin-containing foods or natural medicines. These may have an influence on the melatonin metabolism in vivo by induction or inhibition of enzymes important in its metabolism. As was found in experiments conducted by Wang and co-investigators [70], MFCs isolated from *A. dahurica*, phellopterin, bergapten and xanthotoxin, markedly inhibited CYP isoforms (CYP1A1, CYP1A2, CYP1B1 and CYP2C19) in human liver microsomes (HLMs). In humans, CYP1A2 plays a dominant role in melatonin metabolism. Target compounds were tested and showed strong inhibitory effects towards 1A1 and 1A2 isoforms of CYP, with residual activity of less than 20%. Moderate to strong inhibitory effect was observed in CYP1B1 isoenzyme, and it was weak against CYP2C19. All of these aspects together have an influence on melatonin metabolism. The CYP1A2 isoform was strongly inhibited by the tested compounds (*K_i_* values: 6.34, 5.34 and 18 nM, respectively). Also, an in vivo pharmacokinetic study resulted in 12- to 4-fold increase in melatonin AUC and C_max_ in human subjects intaking 21 mg of melatonin daily and an extract from *A. dahurica* simultaneously.

#### 4.6.2. Effects on Caffeine Metabolism

Since both caffeine and furanocoumarins are metabolized in humans by the same hepatic isoenzyme, CYP1A1/2, products containing caffeine and furanocoumarins administered previously or concomitantly (e.g., in the daily diet) may interact. In the study of Alehaideb et al. [71], the influence of dietary intake of MFC-containing food on caffeine metabolism was investigated. The inhibition of caffeine metabolism was observed (compared with the untreated group of male human volunteers aged 21–30 years old) depending on the MFC content in the diet. The in vitro study additionally showed that individual MFCs were potent inhibitors of caffeine N-demetylation (IC50 amounts for xanthotoxin, bergapten and isopimpinellin were 0.09, 0.13 and 0.29 µM, respectively). CYP1A2 inhibition by individual MFCs was dose and time dependent. The mechanism of inhibition was investigated in HLMs using ^14^C-labeled xanthotoxin. It was confirmed that restoration of the CYP1A2 isoenzyme activity was possible only by isoenzyme re-synthesis (long-term process lasting over 3 h), which confirmed the irreversible mechanism of inhibition. As a result, it was found that simultaneous administration of MFCs containing food products can increase concentrations of drugs that are substrates for CYP1A2 isoenzyme in humans and may have health consequences.

In the study of Alehaideb and Matou-Nasri [72], it was found that consumption of MFCs that are the inhibitors of P450 (CYP) isoenzymes (including CYP1A2), xanthotoxin, bergapten and isopimpinellin, exert a potentiating (synergistic) effect when ingested in traditional Chinese medicine extracts. It is important because it has an influence on xenobiotic metabolic processes when consumed. The influence of these three MFCs on CYP1A2-mediated caffeine metabolism inhibition was evaluated applying different doses of these compound mixtures in in vivo studies (tested on healthy male volunteers aged 20–35 years) via ingesting *Ammi majus* and *A. archangelica* aqueous extracts (decoctions). In this study, inhibitory potencies of pure MFCs on the CYP1A2 isoenzyme were also determined (IC50 values were 61.6, 68.8, and 277.0 nM for xanthotoxin, bergapten and isopimpinellin, respectively). The calculated RPF (relative potency factor) values were 1.00, 0.88, and 0.27, respectively. All of these factors together confirmed the influence of MFC consumption on drug metabolism, which should be taken into consideration when natural medicines are ingested.

#### 4.6.3. Inhibition of Xenobiotic Metabolism

The human P450 (CYP) enzyme family, including CYP1A1, CYP1A2, and CYP1B1, is involved in the metabolism, via an aromatic hydrocarbon receptor (AhR)-mediated signaling pathway, of a large number of procarcinogenic agents, especially polycyclic aromatic hydrocarbons such as, e.g., benzo[α]pyrene (BαP). Joshi et al. [73] assessed the CYP inhibitory effects of various compounds derived from natural products, including MFCs bergapten, isopimpinellin and phellopterin. The first two compounds were found to be the strongest inhibitors of the CYP1A1 enzyme expressed in HEK-293 cells (derived from human embryonic kidney cells) transfected with the pcDNA3.1/CYP1A1 plasmid. Cells were incubated with different concentrations of compounds for 30 min, and the calculated IC50 values were for bergapten and isopimpinellin, 80 and 20 nM, respectively. Phellopterin inhibited CYP1A1 with a moderate potency of IC50 = 580 nM. The most active MFC, isopimpinellin, was found to have a chemopreventive effect by inhibiting the CYP1A1 isoenzyme and protecting HEK293 cells from BαP-induced toxicity by blocking the metabolism of procarcinogenic agents to active carcinogenic metabolites. This opens up a new chemopreventive strategy that can be used for cancer prevention in cases of xenobiotic poisoning.

### 4.7. Influence of MFCs on Enzyme Activities

#### 4.7.1. Influence on Tyrosinase Activity

Tyrosinase, a copper-containing enzyme, plays a crucial role in melanogenesis, and compounds with the ability to inhibit this enzyme serve as skin-whitening ingredients (e.g., vitamin C, kojic acid or arbutin). MFCs isolated from *A. dahurica* roots, xanthotoxin, bergapten, isopimpinellin, phellopterin, cnidilin and byakangelicol, were evaluated (at a concentration of 25 µM) on their tyrosinase inhibitory activity (kojic acid was used as positive control), and this activity was less than 5% [61].

Two reactions catalyzed by the enzymatic activity of tyrosinase (the hydroxylation of L-tyrosine to L-DOPA and next to DOPA-quinone) are crucial for the start of melanin synthesis (melanogenesis). Lee and Hyun [74] performed experiments on B16F10 melanoma cells with bergapten, xanthotoxin and isopimpinellin as modulators of melanin production. All of the tested compounds increased melanogenesis in B16F10 cells. Bergapten (at concentrations of 6.25, 12.5, and 25 µM) increases the protein levels of the microphthalmia-associated transcription factor (MITF), tyrosinase activity (to 223.7% at 25 µM), tyrosinase-related proteins-1 and 2 (TRP-1 and especially TRP-2) in B16F10 cells (Western blot results). Moreover, bergapten promotes the phosphorylation of Akt at Ser473 and glycogen synthase kinase-3β at Ser9. Bergapten treatment significantly increases the content of β-catenin (protein involved in regulation and coordination of cell–cell adhesion and gene transcription) in the cell cytoplasm and nucleus by reducing the phosphorylated catenin content (p-β-catenin). It was finally concluded that the regulation of melanogenesis by bergapten may be mediated by β-catenin and p38 mitogen-activated protein kinase (MAPK) signaling pathways.

#### 4.7.2. α-Glucosidase and α-Amylase Inhibition

In the study of MFCs, *H. persicum* moellendorfiline significantly inhibited enzyme α-glucosidase (IC50 = 17.9 nM) compared to acarbose (IC50 = 23.5 nM), a clinically used anti-diabetic drug for type 2 diabetes mellitus. Angular MFCs were confirmed to be more potent inhibitors of α-glucosidase than linear ones. The presence of two methoxy groups as in pimpinellin (C-5 and C-6) enhances activity compared to that of isobergapten (C-5); it is less active than sphondin (C-6). Moellendorffiline, the most potent inhibitor, is a dimer of pimpinellin, and dimerization significantly enhances its activity. Among the linear MFCs, bergapten (methoxy at C-5) was more potent than xanthotoxin (C-8). However, isopimpinellin with two methoxy groups (C-5 and C-8) was less active [60].

Phellopterin (1 mg/kg and 2 mg/kg), isolated from *A. dahurica*, was found to enhance insulin sensitivity and lower blood glucose and lipid (triglyceride and total cholesterol) levels in a mice model of diabetes [75] induced by high-fat diet (HFD) and streptozotocin (STZ). Treatment of phellopterin increased the mRNA expression of peroxisome proliferator-activated receptors γ (PPARγ), which is a major mediator of insulin sensitivity. Additionally, pehellopterin promoted adipocyte differentiation in 3T3-L1 preadipocyte cells. Therefore, this MFC could be considered as an important antidiabetic agent.

Quek et al. [63] studied antidiabetic activities of MFC halfordin (0.078–10 mg/mL), which was isolated from the bark of *M. latifolia* (Rutaceae). In vitro α-amylase inhibitory assay was performed. Halfordin showed high α-amylase inhibition (IC50 = 54.53 µg/mL = 197.53 µM). The active site of α-amylase is made up of three important residues: Asp197, Glu233, and Asp300, and amino acid residues including Trp59, Trp58, and Tyr62 are important for the substrate binding to the enzyme. An in silico study confirmed that halfordin formed a high number of molecular interactions with critical amino acid residues of α-amylase. The methoxy groups attached at the benzene moiety of halfordin formed hydrogen bonding interactions with the carboxyl group of Asp300 (catalytic triad residue), as well as the carbonyl groups of Tyr62 and Trp59. The hydroxyl group of residue Thr163 formed additional hydrogen bonding interaction with the methoxy group of this MFC. The aromatic system in halfordin stabilized binding through hydrophobic π-π interactions with Trp58 and Trp59. These interactions were found to be consistent with the interactions of acarbose with α-amylase and could be responsible for the inhibition of this enzyme [15,63].

### 4.8. Central Nervous System (CNS) as the Target for MFCs

#### 4.8.1. Anticonvulsant and Antiseizure Activity

Epilepsy is a chronic neurological disease characterized by unprovoked recurrent epileptic seizures which can be controlled with antiseizure drugs (ASDs). However, 30% of patients experience no benefit from this medication, and new strategies should be tested [76]. In the search of new ASDs to treat epilepsy, the behavioral screening of natural MFCs, bergapten, phellopterin, byakangelicin, byakangelicol and pimpinellin, was performed employing the larval zebrafish pentylenetetrazole model (PTZ) using locomotor measurements [76]. This is a model of tonic–clonic type of seizures caused by the inhibition of the γ-butyric acid type A receptor (GABA_A_). As was investigated before by this research team, xanthotoxin showed clear anticonvulsant activity in mice (ED50 = 219.1 mg/kg) when administrated 60 min before electroconvulsion [20]. These results suggest that the C-8 methoxy group is important for antiseizure activity (bergapten with the C-5 methoxy group, studied before, shows no protective properties). In the present study, angular MFC pimpinellin lowered seizure-like behavior in a concentration-dependent manner from 25 to 60%. This compound decreased power spectral density to 81%, proving to be highly effective against PTZ-induced epileptiform events (in a locomotor assay). GABA-transaminase inhibition was proposed as the possible mechanism of antiepileptic action of active MFCs. Detailed analysis of the behavior of byakangelicin, byakangelicol and phellopterin (all of these compounds possessing aliphatic chains at the C-8 position, and were non-active against GABA-transaminase) and the in silico study led to conclusion that the three non-blocked oxygen atoms in the furanocoumarin structure are essential to the interaction of compounds with the enzyme pocket of GABA-transaminase, and therefore the C-8 position should be non-substituted by an aliphatic chain [76].

In a recent study, tri-MFC halfordin isolated from endemic plant *Halfordia kendack* Guillaumin (Rutaceae) was tested on its antiseizure activity on a zebrafish PTZ-induced seizure model [21]. Halfordin was subjected to locomotor assay, local field potential (LFP), and gene expression assay. It was found that this MFC (20 µM) decreased convulsive-like behavior in the first two assays (41.4% in a locomotor assay and 60% in LFP). Affinity to GABA_A_ was structurally dependent. Halfordin substituted at C-3, C-4 and C-5 positions exerted significant antiseizure activity, although it was lower than that of C-5-substituted pimpinellin. To explain the antiseizure activity of halfordin, expression of 11 genes was studied. Expression of four genes was changed significantly, among them that of the *penka* gene (proenkefalin encoding endogenous opioid proenkephalin a, regulating responses to stress and possessing analgesic function). Its expression was upregulated after halfordin treatment.

#### 4.8.2. Antidepressant Activity

In depression, in both hippocampus and prefrontal cortex (regions engaged in creating and controlling memory and emotions) neuronal and glia atrophy is observed. The effectiveness of therapy in depression is strongly dependent on sex differences. Xanthotoxin influence (administrated at 12.5 mg/kg) on male and female Swiss mice depression-like behavior was examined. It was suggested that this MFC can affect depression-like behavior in a sex-dependent manner (an antidepressant effect was evident only in males) as was analyzed in a forced swimming test (FST). However, the levels of noradrenalin (NA) and serotonin (5-HT) increased in a dose-dependent manner only in the female prefrontal cortex. There was no correlation between monoamine levels in the hippocampus and behavior in the experiments [77]. In a previous study of this research group, xanthotoxin (15 mg/kg) injected with nicotine also reduced depressive-like behavior in male mice only (FST test) [78].

#### 4.8.3. Procognitive Activity–AChE and BChE Inhibition and Anti-Amyloid-β Activity

Inhibition of the activity of enzymes antiacetylcholinesterase (AChE) and butyrylcholinesterase (BChE) is part of treatment of Alzheimer disease (AD), a neurodegenerative disorder which affects cognitive functions in brain. In this disease, the low level of cholinergic neurotransmitters is related to memory impairment and loss of attention. However, the molecular mechanism of AD remains unclear; the treatment is focused, among other aspects, on the restoration of ACh levels after inhibition of enzymes involved in their decomposition [79].

MFCs isolated form *H. mellendorffi*, such as isobergapten, pimpinellin and (*3S*, *4R*)-3-4-epoxypimpinellin, were analyzed on their AChE inhibitory activity (the Ellman method), and it was found that IC50 values for these compounds were 18.1, 21.5, and 22.9 µM, respectively [80], confirming their activity.

MFCs (bergapten and pimpinellin; 20 µg/mL of each) from the roots of *Zosima absinthifolia* Link. (Apiaceae) were assayed for AChE (the Ellman method) and BChE inhibitory activities in in vitro and in silico molecular docking. Pimpinellin was the most active compound against BChE (66.55% of inhibition), and bergapten exerted 31.0% of inhibition. In molecular docking studies, pimpinellin had a −5.78 kcal/mol docking score and two stacking π-π interactions with the phenyl ring of Phe329 of the enzyme. Polar interactions were realized by Ser287, Gln119, and Ser118, and between the pimpinellin molecule and Phe329, Pro285, Leu286, Val288, Trp231, Phe298, and Ala199 residues. It was concluded that most important for inhibitory activity of pimpinellin was the presence of methoxy groups at C-5 and C-6 positions [81].

Furanocoumarins (among them MFCs isopimpinellin and phellopterin) isolated from *Toddalia asiatica* (L.) Lam. (Rutaceae) were evaluated as multifunctional anti-Alzheimer agents. AChE enzyme inhibition (the Ellman method) and amyloid β (Aβ) aggregation inhibition (AChE- and self-induced) were examined in the presence of phellopterin. Phellopterin was the most potent MFC inhibiting the AChE enzyme with IC50 = 38 µM (isopimpinellin IC50 = 41 µM) and AChE-induced and self-induced Aβ aggregation (IC50 = 97 and 95 µM, respectively)–(isopimpinellin; IC50 > 500, 125 µM, respectively).

Also, hydrogen peroxide (H_2_O_2_) and Aβ_1–42_-induced toxicity in human neuroblastoma cells (SH-SY5Y) was attenuated by phellopterin [82]. The neuroprotective effect against oxidative stress in SH-SY5Y cells was determined by MTT assay. Pre-incubation of the cells with phellopterin (0.1; 1.0 and 10 µM) attenuated the cell damage induced by H_2_O_2_ compared to the control group. A molecular docking study suggested that substituents at C-6, C-7 and C-8 positions of the MFC structure were important for anti-AChE and anti-Aβ aggregation activity of phellopterin [82].

Interesting results were obtained in another study, where angular MFCs (isobergapten and pimpinellin) were isolated from *Tetrataenium nephrophyllum* (Apiaceae). It was found that pimpinellin (with two methoxy groups at C-5 and C-6 positions) exerted AChE inhibition with IC50 = 51.2 mg/mL (0.208 µM), and isobergapten with one methoxygroup at C-5 had IC50 = 172.1 mg/mL (0.796 µM). Pimpinellin carried out one hydrogen bond with a residue of HIP440 in the enzyme molecule (in silico docking studies) with a docking score of −7.7 kcal/mol. Moreover, the benzene ring in furanocoumarin engaged π-π stacking with Tyr121. Thus, molecular docking studies showed good agreement with previous experimental results [83].

### 4.9. Effect of MFCs on Lipid Metabolism

In the study of Lamichhane et al. [84], compounds isolated from dried immature fruits of *Poncirus trifoliata* (L.) Raf. (Rutaceae), well-known Korean and Chinese folk medicine, were tested for anti-adipogenic activity in mouse embryonic fibroblasts (3T3-L1 preadipocyte cell line). Lipid accumulation in 3T3-L1 cells was tested using the ORO assay (Oil Red O/microscope observation). Phellopterin showed a significant anti-adipogenic effect by inhibition of adipocyte differentiation and lowering the production of lipids in a dose-dependent manner (at concentrations of 5 and 10 ug/mL of phellopterin, lipid production was reduced to 89.76% and 82.94%, respectively). The key adipogenic markers, such as peroxisome proliferator-activated receptor proteins γ (PPAR-γ), sterol response element binding protein-1 (SREBP-1), CCAAT/enhancer binding protein-α (C/EBP-α), adipocyte-specific lipid binding protein (FABP-4), adipocyte fatty acid binding protein (aP2), lipoprotein lipase (LPL), and leptin, were downregulated; however, these studies were performed only for oxypeucedanin (the most active ingredient of *P. trifoiata*), and not for phellopterin.

### 4.10. MFCs as Hemostatic Agents

The natural Chinese medicine, *T. asiatica* (L.) Lam. bark coumarin, was investigated on its hemostatic activity in new experiments (chemical–fingerprint–pharmacokinetic–pharmacodynamic; CP-PK-PD) conducted by Zhang et al. [85]. Isopimpinellin, pimpinellin, and 5-methoxy-8-hydroxypsoralen were detected in rat plasma using the UPLC-ESI method. In blood samples, prothrombin time (PT), activated partial thromboplastin time (APTT), and fibrinogen (FIB) were analyzed after administration of tested MFCs in an aspirin-induced hemorrhagic rat model. FIB was the most suitable parameter to investigate the hemostatic activity of compounds. Tested compounds exhibited relatively rapid absorption and slow elimination characteristics. Isopimpinellin and pimpinellin were in vivo pharmacologically active in hemostasis through activation of coagulation pathways and the fibrinolytic system. An increase in FIB content was observed (with EC50, in µM, of 1.39 and 2.47, for isopimpinellin and pimpinellin, respectively), indicating that the fibrin system was regulated by these MFCs.

### 4.11. MFCs as Agonists of Bitter Taste Receptors (TAS2Rs)

Bitter taste receptors in the oral taste buds, as peripheral chemoreceptors, mediate taste perception in mammals. The detection of bitter substances is mediated by G protein-coupled receptors belonging to the taste 2 receptor (TAS2R) family that are present in specialized taste receptor cells located on the tongue and in the oral cavity [86]. Human TAS2Rs are able to form homo- and heterodimers, and they can be categorized into four groups, the three receptor “generalists” with extensive agonist spectra comprising TAS2R10, 14, and 46 (each able to respond to about one third of the bitter substances), a number of narrowly tuned receptor “specialists” (that detect few bitter compounds), and the intermediately tuned receptors representing the majority, as well as two receptors TAS2R16 and 38 which exhibit pronounced selectivity for defined classes of chemicals [87]. In recent years, it has been revealed that these receptors (called ectopic receptors) are also expressed in the respiratory, gastrointestinal, genitourinary, cardiovascular, and central nervous systems [43]. They are involved in a variety of biological processes, including muscle regeneration, broncho-dilation/constriction, inflammation, appetite regulation, energy metabolism, and also defense system and immunological response [88]. This makes them potential new targets for disease treatment [86]. The selection of potential agonists of these receptors, synthetic, and also those from natural sources, is constantly in progress.

In the study of Mancuso et al. [89], the activity of bergapten, xanthotoxin, and isopimpinellin, as agonists of TAS2Rs, was investigated. The study was conducted using a qualitative and next a quantitative approach. The experiments were performed using HEK293T cells (ATCC), and the cDNAs for the different *TAS2Rs* were transiently transfected using Lipofectamine2000 (Invitrogen); they were analyzed 24 h after transfection. All tested MFCs responded selectively to TAS2R receptors to varying degrees. Bergapten (methoxy at C-5) was a selective strong agonist of only TAS2R10. Isopimpinellin (methoxy at C-5 and C-8) bound to TAS2R10 and TAS2R14, while xanthotoxin (methoxy at C-8) affected TAS2R10 and TAS2R49. Efficacy against TAS2R10 decreased as follows: 5-MOP > 5,8-MOP > 8-MOP, and the C-5 position of the methoxy group was important for activity. In the presence of a second group at the C-8 position, activity decreased, coming to its lowest level when the methoxy group was only at the C-8 position. Activity against TAS2R14 followed the order of 5,8-MOP > 5-MOP, and xanthotoxin (methoxyl at C-8) was inactive. TAS2R10 and TAS2R 14 were quite “generalist” and tuned by a large number of agonists [87,90]; therefore, it was difficult to find selective agonists for these two receptors. The role of TAS2R49 is scarcely described, and only two compounds (cromolyn and diphenidol, both synthetic compounds) were selected as non-selective agonists [90]. As reported in a study by Mancuso and co-investigators, xanthotoxin is the first identified natural compound capable of activating the TAS2R49 receptor.

In another experiment, potential drug screening for tachycardia treatment was conducted, and among the tested drugs was xanthotoxin, which activated specific TAS2Rs receptors (TAS2R119, TAS2R120, and TAS2R135) on HL-1 cardiomyocytes [44]. Although the exact signaling pathway of TAS2Rs in cardiomyocytes is still inconclusive, researchers agree that TAS2R agonists can mediate the contractile response of the cardiovascular system [44]. There is one from two typical signal transduction pathways of TAS2Rs, namely the Gβγ-PLCβ2/IP3 (G protein βγ subunit–Phospholipase C β2/Inositol triphosphate) pathway (where PLCβ2 is an essential component of the signal transduction), the activation of which, as well as the degradation of cAMP, further increase the intensity of intracellular Ca^2+^ signals, causing Ca^2+^-dependent TRPM5 (*taste-signaling transient receptor potential ion-channel*) channels to open, Na^+^ to influx and depolarize the cell membrane, generation of action potentials and release of ATP, which ensures bitter taste signaling [91]. In addition, the binding of bitter agonists to TAS2Rs generates second messenger molecules such as cAMP, which can alter the state of cardiomyocyte membrane ion channels, especially the opening and closing of Ca^2+^, K^+^, and Na^+^ ion channels, and can regulate the polarization and depolarization of cardiomyocyte membrane potential and action potential generation, thus regulating the activity and contractility of cardiomyocytes [91]. Firing rate statistics of cardiomyocyte EFP signals stimulated by different concentrations of xanthotoxin (1–250 μM) were tested by Qin et al. [44]. They showed that 1 μM of xanthotoxin had almost no effect on the firing rates of cardiomyocytes, while 10–250 μM of xanthotoxin had a significant inhibitory effect. IC50 values were calculated from the nonlinear regression analysis of xanthotoxin (IC50 = 14.68 μM). U73122, an inhibitor of PLCβ2, was used to inhibit the Gβγ-PLCβ2 signaling pathway transduced by taste receptor activation in this experiment to evaluate the obtained results [44]. The bitter taste receptors endogenously expressed in HL-1 cells were verified by RT-PCR and immunofluorescence staining. Then, HL-1 cardiomyocyte-based integrated gustatory sensing array coupling with the microelectrode array (MEA) was first constructed for drug screening and evaluation for tachycardia treatment.

**Figure 4:**
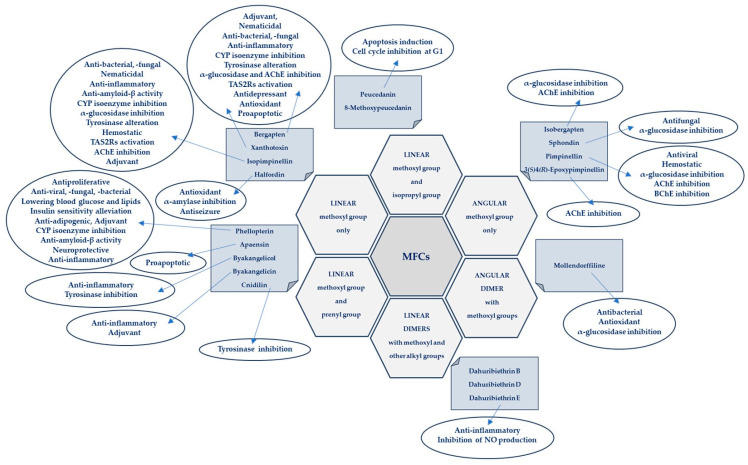
Biological activities of MFCs in relation to the structural type and substituents in the furanocoumarin molecule.

The abovementioned aspects of the recent research findings in MFC studies are summarized in Table S1 (in Supplementary Files). It contains CAS numbers, IUPAC chemical names of the MFCs, molecular formulae and molecular weights of each MFC, and briefly summarizes biological activity and mechanisms of action of each discussed compound. Additionally, Figure 4 presents biological activities of MFCs in relation to the structural type and substituents in the furanocoumarin molecule.

## 5. Structure–Activity Relationships of the Studied MFCs—A Short Summary

On the basis of the obtained results, we can conclude some general observations regarding the structure–activity relationship of the analyzed MFCs. The particular position of the methoxyl group and the count of MFCs may influence the biological activity of linear MFCs. It was especially found when MFCs such as xanthotoxin, bergapten, and isopimpinellin as agonists of the bitter taste receptors were studied [89], where the specificity of the receptor target was also related to the position of the methoxy group in the furanocoumarin scaffold. The C-8 methoxy group was important for antiseizure activity of xanthotoxin, and bergapten with the C-5 methoxy group showed no protective properties [76]. In the case of α-glucosidase inhibitory activity [60] and antibacterial activity of MFCs [14], it was also important whether the molecule was in a linear or in an angular shape. Angular structures were more active (pimpinellin vs. isopimpinellin) and dimers were more active than monomers (moellendorffiline vs. pimpinellin).

The isopenthenyl group on the furanocoumarin skeleton increase their lipophilicity and the possibility of passage of the molecule through the bacterial membrane to its target site. Phellopterin possesses this group at the C-5 position. Double oxygenated substituents at C-5 and C-8 are necessary for antibacterial activity of linear furanocoumarins, such as in the case of isopimpinellin and phellopterin [49]. The latter one has a prenyl group, which increases lipophilicity, and therefore phellopterin was more active against bacterial strains than isopimpinellin [50]. Similar behavior was observed in the case of the adjuvant properties of byakangelicin, which increase the content of the co-ingested drugs in the brain by enhancing their penetration due to they own lipophilicity [67].

The presence of methoxy groups at C-8 and C-5 in the structure of furanocoumarin and the angular type of this structure are also beneficial for antifungal activity [53]. The analysis of the influence of methoxyl groups on the inhibition of AChE activity by angular MFCs indicated that pimpinellin (with two methoxy groups at C-5 and C-6 positions) exerted three-fold higher activity on AChE inhibition than isobergapten with one methoxy group at the C-5 position [86]. The angular MFC pimpinellin (with two methoxy groups) was significantly (two-fold) more active compared to bergapten (linear with C-5 methoxy group) in the BChE inhibitory assay [81].

The furan ring fused at the C-6 and C-7 positions of the coumarin scaffold is necessary, and also the 5-methoxy group contributes greatly to reducing histamine release; however, the isopentane-derived groups in the molecule likely reduce histamine release as well [56]. Moellendorffiline (dimer of pimpinellin) exerts higher antioxidant activity compared to the monomeric compound, pimpinellin [60].

Inhibition of GABA-transaminase has been proposed as a possible mechanism for the antiepileptic effects of active MFCs. Analysis of the behavior of byakangelicin, byakangelicol and phellopterin (all these compounds have aliphatic chains at the C-8 position and are not active against GABA-transaminase) and in silico study showed that the three unblocked oxygen atoms in the MFC structure are necessary for the interaction of compounds with the enzyme pocket of GABA-transaminase. Therefore, if the compounds are to be active, the C-8 position cannot present an aliphatic chain [76].

All these observations are important, but many of them require additional, in-depth research, and this knowledge may be important when designing drugs based on MFC. It seems particularly interesting to examine the interaction of various substituents (methoxy, hydroxyl, isopropyl and prenyl groups) in the molecule, the position and number of which can modify activity. It should be taken into account that the impact of substituents may also depend on the selected research model and various factors, including physicochemical ones, and in the case of multi-component drugs, on the impact of co-existing molecules. All of this together makes this aspect even more difficult to explore, but it can also be an exciting challenge.

The biological activities of MFCs discussed in this article in relation to the structural type and substituents in the furanocoumarin molecule are briefly presented in Figure 4.

## 6. Toxicity and Safety Studies

As consumption of natural medicines continues to increase, attention should be paid to the potential risks associated with ingested bioactive compounds. When developing new therapeutic strategies, including those related to natural medicines, safety of use (including toxicology) and assessment of side effects are an extremely important part of the related procedures. It has been demonstrated that toxicity induced by herbal medicines may result from the biotransformation of their components into electrophilic, reactive metabolites that can covalently bind to important macromolecules in the body. The complicated relationship between the active ingredients of natural drugs, their detoxification mechanisms and potential risks was highlighted in a recently published review article of Wen and Gorycki [92]. Furanocoumarins were also mentioned, metabolized to reactive toxic forms such as epoxides, cis-2-enedialdehyde, γ-ketoenal, and detoxified, the bioactivation of which may lead to the inactivation of drug metabolizing enzymes (including CYP isoenzymes); it clinically manifests itself through interactions and side effects when taking plant ingredients and synthetic drugs.

Bioactivation of components of herbal extracts and the associated toxicity are largely governed by the complex composition of said extracts, so understanding the complexity of natural medicines is of great importance [93]. As was underlined by Kharaman and co-investigators [93], the toxicity of a single compound can be reduced by interactions with accessory molecules present simultaneously in natural medicines. The different biological activities of these reactive metabolites can be viewed as a function of their reactivity, selectivity, concentration, and exposure time, as well as the pro-oxidant/antioxidant balance in cells [92].

A new approach to assessing the risk associated with the consumption of drugs and herbal preparations was proposed by Wang et al. [94] on the example of multi-component preparations of *A. dahurica* containing MFCs. It is worth undertaking and constantly developing this important aspect of the safety of natural medicines and functional foods [95,96].

## 7. Conclusions and Future Perspectives

Network pharmacology [97] and in silico studies allow, in many cases, the finding of compounds with the desired biological activity or prediction of their potential genotoxicity, and these approaches were used in MFC research [95]. The obtained results were then successfully verified in targeted in vitro and in vivo experiments.

In a recently applied PAMPA (parallel artificial membrane permeability assay) study, the effect of complex mixtures and their synergistic influence on the absorption of certain MFCs (such as, e.g., bergapten and isopimpinellin) was successfully investigated [28,29].

An interesting approach is to use new animal models, such as zebrafish, to study the CNS-targeted properties of MFCs [21]. This model also has great potential for studying many other possible biological activities of these compounds. On another note, introducing new models into MFC research, such as 3D cell cultures/organoids, could provide an opportunity to minimize the use of experimental animals and increase the accuracy of studies designed to be similar to research on the human body.

The use of MFCs as adjuvants in anticancer and antibacterial therapies is a promising direction, allowing the reduction in therapeutic doses and the possibility to avoid some side effects occurring during anticancer treatment, as well as restoring the sensitivity of microorganisms to conventional therapies [19,50,65].

The study of non-UV-activated MFC molecules from the psoralen group is an interesting alternative to assessing the anti-proliferative and pro-apoptotic effects on some multi-resistant cancer cell lines without the need to use UV radiation [16,17]. Given the specific sensitivity of some cancer cells, this may be the goal of extensive research in this area. It is worth undertaking further research on MFC metabolic pathways in animals and humans, as well as metabolism by intestinal microflora and its impact on MFC bioavailability. Key aspects also include testing the toxicity and safety of MFCs both in vitro and in vivo, as well as examining the interactions of these compounds with food ingredients and drugs.

Research on agonists attained from the MFC group of tissue-specific bitter taste receptors (TAS2Rs) [44,89], once constructed appropriate experimental models are available, may constitute an interesting direction in the development of new drugs based on MFCs, as well as targeted therapies using them.

Finally, new applications of MFCs (bergapten and pimpinellin), such as environmentally friendly and effective anti-corrosion materials, have recently been successfully proposed [98].

**Figure 5:**
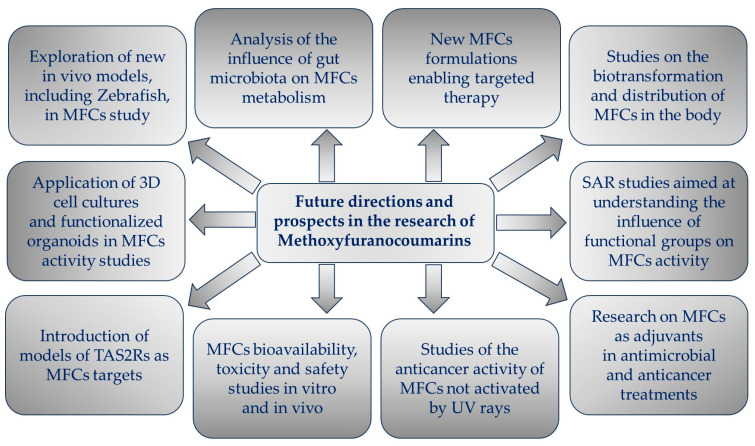
Future directions and prospects in the research of methoxyfuranocoumarins (MFCs).

All of these are exciting prospects of the multidirectional development in the field of MFC research (Figure 5).

In summary, MFCs are molecules that are constantly being rediscovered, having promising potential for future applications that will hopefully have an impact on human well-being and medical care, and therefore deserve special attention in future, large-scale research, including interdisciplinary studies.

## Data Availability

All of the associated data are available within the manuscript.

## Supplementary Materials

The following supporting information can be downloaded at: https://www.mdpi.com/article/10.3390/cimb46010055/s1, Table S1 contains CAS numbers, IUPAC chemical names of MFCs, molecular formulae and molecular weights of each MFC, and briefly summarizes activity and mechanisms of action of each discussed molecule.
