# Supplementary material for: Methoxyfuranocoumarins of Natural Origin–Updating Biological Activity Research and Searching for New Directions—A Review
Source: Curr Issues Mol Biol. 2024 Jan 19;46(1):856–83. doi: 10.3390/cimb46010055 (PMC10813879; doi:10.3390/cimb46010055)
Supplement: Supplementary file 1 [file cimb-46-00055-s001.zip › cimb-2827441-supplementary.pdf]

Table S1 - IUPAC names, CAS numbers, molecular formulas and molecular weights, biological properties, mechanisms of action and experimental models studied in research of MFCs*

No	Compound / CAS No<br>Molecular formula / wt.	IUPAC name	Biological activity<br>[Ref.]	Effects		Model studied
				Upregulation/Stimulation	Downregulation/Inhibition	Cell/Tissue/Organism
**PSORALENE-TYPE**						
**Methoxyl group(s) only**						
**1**	Bergapten<br>(5-methoxypsoralen)<br>CAS 484-20-8<br>$C_{12}H_8O_4$ / 216.189 Da	4-Methoxy-7H-furo[3,2-g]chromen-7-one	Antibacterial [49]<br>Antifungal [53]<br>Nematicidal [54]<br>Anti-inflammatory [56]<br>Antioxidant [61]<br>Adjuvant [19,66]*<br>Proapoptotic [17]# [69]^<br>CYP isoenzymes inhibition [70,71,72,73]<br>Tyrosinase alteration [61,74@]<br>α-glucosidase inhibition [60]<br>BChE inhibition [81]!<br>TAS2R10 agonist [89]	# apoptosis induction (caspases -8, -9, -3 activation; Bax protein expression)<br>@ (increase β-catenin content, and Tyrosinase activity up to 223.7%)	* Enhancement of inhibitory effect of 5FU<br># (AKT phosphorylation and downregulation of AKT/PKB; Bcl-2)<br>^ Cell cycle inhibition in G1 (47.3 %) after 48 h<br>! IC50=31 μM (concentr. 20 μg/mL)	* SMMC-7721 human hepatocellular carcinoma – in vitro<br># osteosarcoma Saos-2 (40.05 μM)<br># colon cancer cells (HT-29 and SW620)<br>^ HL60 cells – in vitro<br>@ B16F10 melanoma – in vitro<br>! Ellman method
**2**	Xanthotoxin<br>(8-methoxypsoralen)<br>CAS 298-81-7<br>$C_{12}H_8O_4$ / 216.189 Da	9-Methoxy-7H-furo[3,2-g]chromen-7-one	Adjuvant [19]<br>Antiviral [47]<br>Antifungal [52,53]<br>Nematicidal [54]<br>Antioxidant [61]<br>Proapoptotic [16]*[69]^<br>CYP isoenzymes inhibition [70,72,73]<br>Tyrosinase alteration [61,74@]<br>Anticonvulsant [76]<br>Antidepressant [77,78]#<br>α-glucosidase inhibition [60]<br>TAS2R10 and 49 agonist [89]<br>TAS2R110,120,135 agonist [44]!	* apoptosis induction (caspases -8, -9, -3 activation; alteration Bax protein expression)<br># (NA and 5-HT level in female mice hippocampus)	* decreased Bcl-2 and phosphoroAkt-thr308) PI3K/AKT<br>^ Cell cycle inhibition in G1 (44.2 %) after 48 h<br># (12 mg/kg and 15mg/kg – decreasing depressive-like behavior in male mice)<br>! Gβγ-PLCβ2/IP3 pathway	* neuroblastoma cells: SK-N-AS (IC50=56.3 μM)<br>* colon cancer cells: SW620 (IC50=88.5 μM) - in vitro<br>^ HL60 cells - in vitro<br># Male and female Swiss mice - in vivo<br># B16F10 melanoma – in vitro<br>! HL-1 cardiomyocytes

**3**	5-methoxy-8-hydroxypsoralen (8-hydroxybergapten) CAS 1603-47-0 $C_{12}H_8O_5$ / 232.189 Da	9-Hydroxy-4-methoxyfuro[3,2-g] chromen-7-one	Antioxidant [62]^ Anti-proliferative [62]*	-	^ moderate DPPH scav. act. ^ strong ABTS•+ scav. act. * (IC50 = 7.46 μM IC50 = 13.48 μM)	^ EC50 = 45.24 μM ^ EC50 = 17.86 μM * (HepG2 cells HeLa cells) – in vitro
**4**	Isopimpinellin (5,8-dimethoxypsoralen) CAS 482-27-9 $C_{13}H_{10}O_5$ / 246.215 Da	4,9-Dimethoxy-7H-furo[3,2-g]chromen-7-one	Adjuvant [19,50] Antibacterial [50, 51] Antifungal [52,53] Nematicidal [54] Anti-inflammatory [55]# [56] [56] * Adjuvant [50] Anticonvulsant [65]^ CYP isoenz. inhib. [71,72,73] Tyrosinase alteration [61,74@] α-glucosidase inhibition [60] Anti-amyloid-β activity [82] Hemostatic [85] TAS2R10 and 14 agonist [89]	* (NF-κB) ^ (additive anticonvulsant effects of phenobarbital and valproate)	# (PI3K inhibition AKT phosphorylation) * (TNFα; IL-1β; IL-4)	* (RBL-2H3 cells) – in vitro ^ (mouse MES model) - in-vivo @ B16F10 melanoma – in vitro
**5**	Halfordin (3,4,5-trimethoxypsoralen) CAS 18646-71-4 $C_{14}H_{12}O_6$ / 276.241 Da	4,5,6-Trimethoxy-7h-furo[3,2-g]chromen-7-one	Antioxidant [62]* α-amylase inhibition [63]@ Antiseizure [21]#	# upregulation of *penca* gene	* 88.84 % inhibition @ IC50=197.53 μM (= 54.53 μg/mL)	* β-carotene bleaching assay [% of inhibition]
**Methoxyl and isopropyl groups**			**Biological activity [Ref.]**	**Upregulation/Stimulation**	**Downregulation/Inhibition**	**Cell/Tissue/AOrganism**
**6**	Peucedanin (oreoselone methyl ether) CAS 133-26-6 $C_{15}H_{14}O_4$ / 258.269 Da	2-Isopropyl-3-methoxy-7H-furo[3,2-g]chromen-7-one	Proapoptotic [69]	Apoptosis induction	Cell cycle inhibition in G1; 69.1 % after 24 h 58.6 % after 48 h (concentr. 100 μg/mL)	HL60 cells PBL cells – in vitro
**7**	8-Methoxypeucedanin CAS 76520-50-8 $C_{16}H_{16}O_5$ / 288.295 Da	2-Isopropyl-3,8-dimethoxy-7H-furo[3,2-g]chromen-7-one	Proapoptotic [69]	Apoptosis induction	Cell cycle inhibition in G1; 66.1 % after 24 h 51.9 % after 48 h (concentr. 100 μg/mL)	HL60 cells PBL cells – in vitro

Methoxyl and prenyl groups			Biological activity [Ref.]	Upregulation/Stimulation	Downregulation/Inhibition	Cell/Tissue/AOrganism
**8**	Phellopterin<br>(5-methoxyimperatorin)<br>CAS 2543-94-4<br>$C_{17}H_{16}O_5$ / 300.306 Da	4-Methoxy-9-[(3-methyl-2-buten-1-yl)oxy]-7H-furo[3,2-g]chromen-7-one	Antiviral [45, 47]<br>Antibacterial [49]<br>Antibacterial [50]<br>Anti-inflammatory [58]^ [59]*<br>Adjuvant [64]# [50]&<br>Anti-proliferative [62]$<br>CYP isoenzyme inhib. [70,71]<br>Insulin sensitivity alteration [75]@<br>Lowering blood glucose [75]<br>Lowering blood lipids [75]<br>Anti-amyloid-β activity [82]<br>Neuroprotective [82]<br>Anti-adipogenic [84]!	* (SIRT1 protein)<br><br>@ (mRNA expression of PPARγ; adipocite differentiation promotion)	^ (IgE)<br>* (ICAM-1 and INF-γ)<br><br>#(P-glycoprotein inhibition; IC50 = 32 μg/mL)<br><br>$ (IC50 = 7.49 μM)<br>@ 3T3-L1 preadipocyte cells<br><br>! Downregulation;<br>- adipocyte-specific protein<br>- fatty acids binding proteins<br>- lipoprotein lipase<br>- leptin	^ HaCaT in vitro, C57BL/6 male mice<br>- in vivo<br>* (C57BL/6J mice)<br># SA1199B and SA-K2191 (*S. aureus* with NorA and MrsA efflux proteins)<br>$ (HepG2 cells)<br>@ (3Ts-L1 preadipocyte cells) – in vitro
**9**	Cnidilin<br>(Isophellopterin = 8-methoxyisoimperatorin)<br>CAS 14348-22-2<br>$C_{17}H_{16}O_5$ / 300.306 Da	5-(Isopentenyl-oxy)-8-methoxypsoralen; 9-Methoxy-4-[(3-methyl-2-buten-1-yl)oxy]-7*H*-furo[3,2-*g*][1]benzopyran-7-one	Tyrosinase inhibition [61]	-	Inhibition less than 5%	-
**10**	Apaensin<br>(8-methoxypabulenol)<br>CAS<br>$C_{17}H_{16}O_5$ / 319.281 Da	4-{[(2S)-2-Hydroxy-3-methyl-3-buten-1-yl]oxy}-8-methoxy-7H-furo[3,2-g]chromen-7-one	Proapoptotic [68]	Nur77 receptor and JNK and p38 (MAPK)<br>(Nur77-Bcl-2 activation)<br>Bax	-	NIH-H460 lung cancer cells – in vitro
**11**	Byakangelicin<br>CAS 19573-01-4<br>$C_{17}H_{18}O_7$ / 334.32 Da	9-(2,3-Dihydroxy-3-methylbutoxy)-4-methoxy-7H-furo[3,2-g][1]benzopyran-7-one	Anti-inflammatory [56] *<br>Adjuvant [67]^	* (NF-κB)	* (TNFα; IL-1β; IL-4)<br><br>^ (TNFα; IL-1β)	* basophilic leukaemia RBL-2H cells – in vitro<br>^ LPS-induced neuro-inflammation model in vivo (B57/BL6 mice)
**12**	Byakangelicol<br>CAS 61046-59-1<br>$C_{17}H_{16}O_6$ / 316.31 Da	9-(3,3-Dimethyl-2-oxiranyl)methoxy]-4-methoxy-7H-furo[3,2-g][1]benzopyran-7-one	Anti-inflammatory [56] *<br>Tyrosinase inhibition [61]	* (NF-κB)	* (TNFα; IL-1β; IL-4)	* basophilic leukaemia RBL-2H cells<br>- in vitro

			Biological activity [Ref.]	Upregulation/Stimulation	Downregulation/Inhibition	Cell/Tissue/Organism
**ANGELICIN-TYPE**						
**Methoxyl groups only**						
**13**	Isobergapten<br>CAS 482-48-4<br>$C_{12}H_8O_4$ / 216.189 Da	5-Methoxy-2H-furo[2,3-h]chromen-2-one	α-glucosidase inhibition [60]*<br>AChE inhibition [80#, 83@]	-	* weak inhibition<br># IC50 = 18.1 μM<br>@ IC50 = 0.796 μM<br>(= 172.1 μg/mL)	#, @ Ellman method
**14**	Sphondin<br>CAS 483-66-9<br>$C_{12}H_8O_4$ / 216.189 Da	6-Methoxy-2H-furo[2,3-h]chromen-2-one	Antiviral [48]*<br>Antifungal [53]#<br>α-glucosidase inhibition [60]	-	* Degradation of HBx hepatitis B protein<br>* Inhibition of expression hepatitis B surface antigen (HBsAg)	* HepG2 cells<br>* mouse model<br># Fungi, yeasts
**15**	Pimpinellin<br>CAS 131-12-4<br>$C_{13}H_{10}O_5$ / 246.215 Da	5,6-Dimethoxy-2H-furo[2,3-h]chromen-2-one	Antibacterial [14, 49]<br>Antifungal [53]<br>Anti-inflammatory [56]<br>α-glucosidase inhibition [60]<br>AChE inhibition [80#, 81, 83*]<br>BChE inhibition [84]^<br>Hemostatic [85]	-	# IC50 = 21.5 μM<br>* IC50 = 0.208 μM<br>(= 51.2 μg/mL)<br>^ IC50 = 66.55%<br>(concentr.20 μg/mL)	# * ^ Ellman method, and in silico study
**16**	3*(S)*4*(R)*-Epoxypimpinellin<br>CAS – not available<br>$C_{13}H_{10}O_6$ / 262.047 Da	(3*S*, 4*R*)-3,4-epoxy-5,6-dimethoxy-2H-furo[2,3-h]chromen-2-one	AChE inhibition [80]	-	IC50 = 22.9 μM – moderate	Ellman method
**DIMERS**			**Biological activity [Ref.]**	**Upregulation/Stimulation**	**Downregulation/Inhibition**	**Cell/Tissue/Organism**
**Methoxyl groups**						
**17**	Moellendorffiline<br>CID: 128785<br>CAS 105099-87-4<br>$C_{26}H_{20}O_{10}$ / 492.431 Da	11,12,13,14-Tetramethoxy-5a,5b,12b,12c-tetrahydrofuro[2'',3'':7',8']chromeno[4',3':3,4]cyclobuta[1,2-c]furo[2,3-h]chromene-5,6-dione	Antibacterial [14]*<br>Antioxidant [60]#<br>α-glucosidase inhibition [60]^	-	* (MIC=15.6-62.50 μg/mL)<br># IC50 = 0.1 μM<br>^ (IC50 = 17.9 nM)	* Bacterial strains<br># DPPH assay

Methoxyl and other alkyl groups			Biological activity [Ref.]	Upregulation/Stimulation	Downregulation/Inhibition	Cell/Tissue/Animal
**18-23**	Dahuribiethrins A - G<br>CAS – not available<br>A; B; D; E; F; G;<br>$C_{33}H_{31}O_{11}$ / 603.1840;<br>603.1860; 603.1868;<br>603. 1853; 603.1853;<br>603.1850	Active dahuribiethrins; B, D, E	Anti-inflammatory [13]	-	Inhibition of NO production;<br>IC50 values;<br>B = 9.6 μM<br>D = 8.8 μM<br>E = 9.2 μM<br>(positive control indomethacin;<br>IC50 = 23.6 μM)	In vitro inflammation model in LPS-stimulated RAW264.7 macrophages

Abbreviations: **DPPH (2, 2-Diphenyl-1-picrylhydrazyl); ABTS**$^{+}$ **(2, 2′-Azinobis-(3-ethylbenzothiazoline-6-sulfonate) radical cation);**

*** CAS numbers, IUPAC names, molecular formula and weight of each MFCs were assessed on-line form Databases; ChemSpider, PubChem, and SciFinder CAS.**

The references numbers in the Table S1 are consistent with the numbering in the main text of the manuscript.

Symbols; *; ^; #; @; !; &; $ represent connection of the cited references (presenting biological activity studies) and the content in the columns regarding described effects (downregulation/inhibition and/or upregulation/activation) and also with the experimental model used in the study.
